# Acyloxyacyl hydrolase promotes pulmonary defense by preventing alveolar macrophage tolerance

**DOI:** 10.1371/journal.ppat.1011556

**Published:** 2023-07-27

**Authors:** Xiaofang Cheng, Wei Jiang, Yeying Chen, Benkun Zou, Zhiyan Wang, Lu Gan, Zeling Xiao, Changshun Li, Cheng-Yun Yu, Yimeng Lu, Zeyao Han, Jiashun Zeng, Jie Gu, Tianqing Chu, Mingsheng Fu, Yiwei Chu, Wenhong Zhang, Jianguo Tang, Mingfang Lu

**Affiliations:** 1 Department of Trauma-Emergency & Critical Care Medicine, Shanghai Fifth People’s Hospital, Department of Immunology, Key Laboratory of Medical Molecular Virology (MOE, NHC, CAMS), School of Basic Medical Sciences, Shanghai Institute of Infectious Disease and Biosecurity, Fudan University, Shanghai, China; 2 Department of Rheumatology and Immunology, the Affiliated Hospital of Guizhou Medical University, Guiyang, China; 3 Department of Pulmonary Medicine, Shanghai Chest Hospital, Shanghai Jiao Tong University, Shanghai, China; 4 Department of Thoracic Surgery, Zhongshan Hospital, Fudan University, Shanghai, China; 5 Department of Gastroenterology, Shanghai Fifth People’s Hospital, Fudan University, Shanghai, China; 6 Innovative Center for New Drug Development of Immune Inflammatory Diseases, Ministry of Education, Shanghai, China; 7 Shanghai Huashen Institute of Microbes and Infections, Shanghai, China; University of Pennsylvania, UNITED STATES

## Abstract

Although alveolar macrophages (AMs) play important roles in preventing and eliminating pulmonary infections, little is known about their regulation in healthy animals. Since exposure to LPS often renders cells hyporesponsive to subsequent LPS exposures (“tolerant”), we tested the hypothesis that LPS produced in the intestine reaches the lungs and stimulates AMs, rendering them tolerant. We found that resting AMs were more likely to be tolerant in mice lacking acyloxyacyl hydrolase (AOAH), the host lipase that degrades and inactivates LPS; isolated *Aoah*^*-/-*^ AMs were less responsive to LPS stimulation and less phagocytic than were *Aoah*^*+/+*^ AMs. Upon innate stimulation in the airways, *Aoah*^*-/-*^ mice had reduced epithelium- and macrophage-derived chemokine/cytokine production. *Aoah*^*-/-*^ mice also developed greater and more prolonged loss of body weight and higher bacterial burdens after pulmonary challenge with *Pseudomonas aeruginosa* than did wildtype mice. We also found that bloodborne or intrarectally-administered LPS desensitized (“tolerized”) AMs while antimicrobial drug treatment that reduced intestinal commensal Gram-negative bacterial abundance largely restored the innate responsiveness of *Aoah*^*-/-*^ AMs. Confirming the role of LPS stimulation, the absence of TLR4 prevented *Aoah*^*-/-*^ AM tolerance. We conclude that commensal LPSs may stimulate and desensitize (tolerize) alveolar macrophages in a TLR4-dependent manner and compromise pulmonary immunity. By inactivating LPS in the intestine, AOAH promotes antibacterial host defenses in the lung.

## Author summary

AOAH is the host lipase that degrades and inactivates Gram-negative bacterial lipopolysaccharides (LPSs). AOAH is required for recovery from LPS-induced macrophage tolerance. Expressed in the gut, AOAH inactivates microbiota-derived LPS. In this study we found that AOAH-deficient mice were less able to contain pulmonary *Pseudomonas aeruginosa* infection than were control wildtype mice. Alveolar macrophages (AMs) from *Aoah*^*-/-*^ mice were hypo-responsive to innate stimulation and they had reduced phagocytic activity. In addition, *Aoah*^*-/-*^ AMs had metabolic changes characteristic of tolerant macrophages as well as increased cell-surface expression of MHC II and co-stimulatory molecules, findings suggesting that they had been stimulated *in situ*. Treating *Aoah*^*-/-*^ mice with p.o. neomycin normalized AMs’ innate responsiveness while intrarectal LPS administration tolerized AMs. We conclude that AOAH regulates pulmonary mucosal immunity in part by inactivating LPS in the gut. This study sheds light on a previously unappreciated mechanism that regulates pulmonary immune defense via the gut-lung axis.

## Introduction

Residing at air-liquid interfaces, alveolar macrophages (AMs) are on the front line of host defense against inhaled microbial pathogens [1–6]. In addition to phagocytosing and killing microbes directly, AMs secrete inflammatory cytokines and chemokines to recruit neutrophils to eliminate pathogens [7–9]. Mice lacking AMs or having hypo-responsive AMs have reduced neutrophil recruitment and are unable to control infections in the lung [7,10].

After macrophages respond to sensing a low dose of a Microbe-Associated Molecular Pattern (MAMP), they usually become hypo-responsive to subsequent MAMP stimulation [11]. This phenomenon, often called “tolerance”, is believed to protect the host from damage caused by excessive or prolonged inflammation, yet the reduction in inflammatory responsiveness may increase susceptibility to secondary infections [12]. AMs isolated from the lungs of experimental septic animals had reduced responses to *ex vivo* LPS stimulation [13–16] as well as decreased phagocytic and bactericidal activity [17,18], and septic animals were more susceptible to pulmonary infections [15]. Notably, after the resolution of respiratory influenza virus infection, AMs remained tolerant for several months, with reduced NF-κB activation and chemokine production upon MAMP re-stimulation [7]. After secondary bacterial challenge, post-infection mice had reduced neutrophil recruitment and significantly increased bacterial burden in the lungs suggesting that AM-derived chemokines and neutrophil recruitment are indispensable for pulmonary defense [7].

Acyloxyacyl Hydrolase (AOAH) is a host lipase that inactivates LPS by removing the secondary fatty acyl chains from the lipid A moiety [19]. Although there are many mechanisms that can dampen LPS stimulation *in vivo*, AOAH is required to detoxify LPS in tissues [20–25]. Importantly, AOAH shortens the duration of endotoxin tolerance. After exposure to a small intraperitoneal dose of LPS, *Aoah*^*-/-*^ peritoneal macrophages remained tolerized for months and *Aoah*^*-/-*^ mice were more susceptible to *E*. *coli* challenge than were wildtype mice [12]. The persistence of fully acylated, bioactive LPS prevented macrophages from regaining innate responsiveness [26]. Thus, continuous LPS stimulation keeps macrophages in a tolerant state, and LPS deacylation by AOAH is required to restore macrophage homeostasis.

Much evidence has supported the hypothesis that the intestinal microbiota can regulate pulmonary mucosal immunity [27–30]. We previously found that excessive bioactive LPS translocated from the gut into the circulation and reached the lungs in *Aoah*^*-/-*^ mice. Persistent stimulation of alveolar epithelial cells induced tolerance, leading to reduced responses to inhaled allergens and less robust allergic reactions [31]. As both AMs and alveolar epithelial cells are sentinels that contribute to pulmonary defenses, we have now tested whether *Aoah*^*-/-*^ AMs are also tolerant and whether *Aoah*^*-/-*^ mice are more susceptible to infection. We found that AOAH prevents/reduces AM tolerance and increases resistance to subsequent pulmonary infection.

## Results

### *Aoah*^*-/-*^ mice are more susceptible to pulmonary infection induced by *Pseudomonas Aeruginosa*

**Fig 1:**
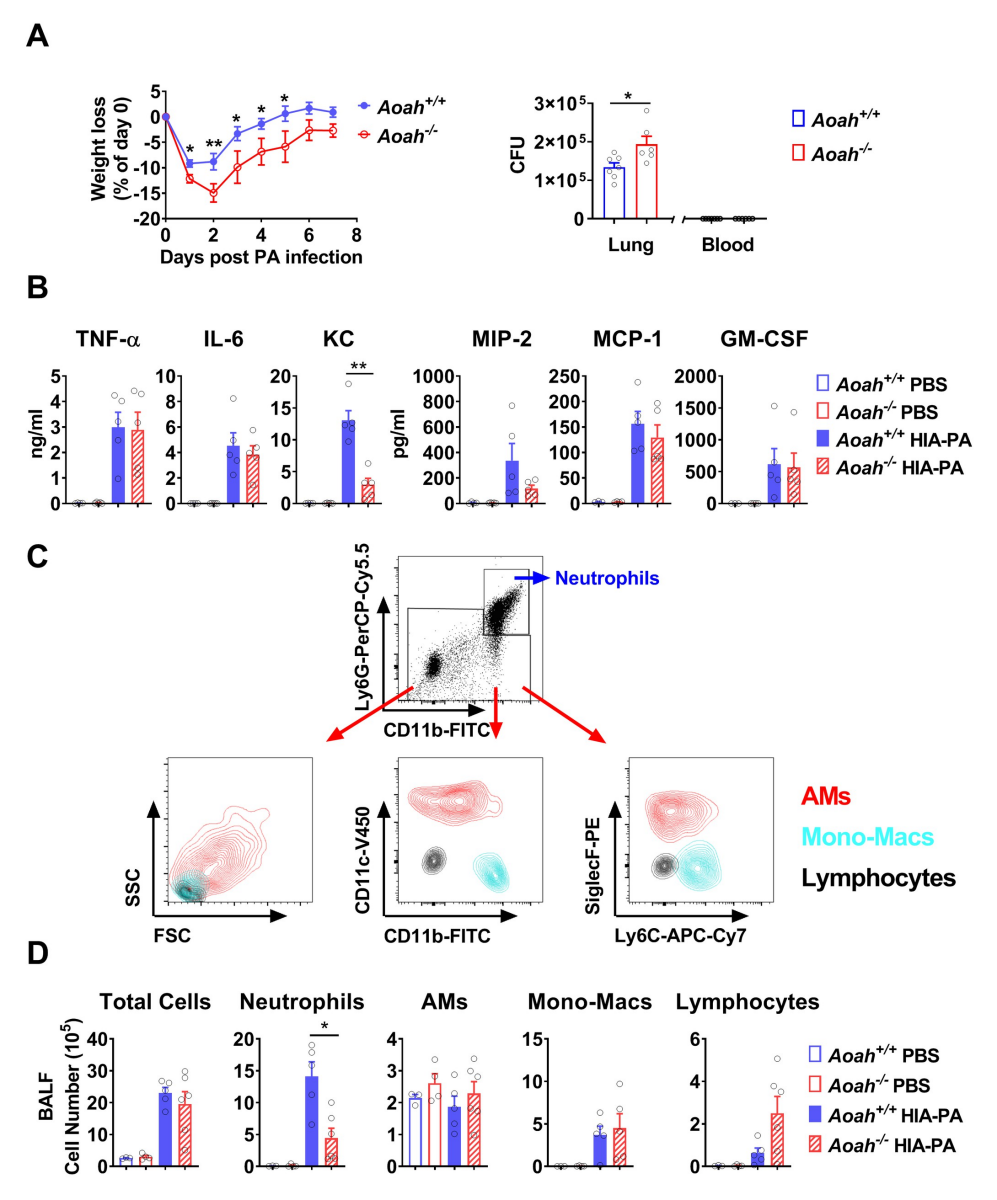
*Aoah*^*-/-*^ mice are more susceptible to pulmonary infection with *Pseudomonas aeruginosa*. (A) Mice were infected with 3 × 10^6^ PA i.n. and their body weights were measured daily for one week. None of the mice died. Data were combined from 3 experiments. n = 10. In another experiment, the lungs and blood were obtained for bacterial load analysis 48 h after infection. No bacteria or fewer than 75 CFUs were recovered from total blood. n = 6 or 7. (B-D) Mice were instilled i.n. with 1 × 10^7^ heated inactivated (HIA) PA. Control mice received PBS i.n. Five h later, cytokines or chemokines in the BALF were analyzed using ELISA. Data were combined from 2 experiments. n = 5 (B). Total cell numbers in BALF were counted. BALF immune cells were identified using FACS: CD11b^+^Ly6G^+^ neutrophils, Ly6G^-^CD11c^+^CD11b^lo^ SiglecF^+^ alveolar macrophages (AMs, red), Ly6G^-^CD11c^lo^CD11b^+^Ly6C^+^ mono-macrophages (monocyte-derived macrophages, cyan), and Ly6G^-^CD11c^-^CD11b^-^SSC^lo^FSC^lo^ lymphocytes (black) (C). BALF immune cell numbers were shown (D). n = 3–6. (A, B and D) Mann-Whitney test was used. *, P < 0.05; **, P < 0.01.

To find out if *Aoah*^*-/-*^ mice are hypo-responsive to inhaled microbes and more susceptible to pulmonary infections, we infected mice with intranasal (i.n.) *Pseudomonas aeruginosa* (PA) [32]. *Aoah*^*-/-*^ mice had greater and more prolonged body weight loss and higher bacterial burden in their lungs than did *Aoah*^*+/+*^ mice (Fig 1A). To test whether the increased susceptibility to PA infection is due to reduced pulmonary innate responses, we instilled heat-inactivated (HIA) PA i.n. and found that PA induced less keratinocyte-derived chemokine (KC or CXCL1, a neutrophil chemokine) production and neutrophil recruitment in *Aoah*^*-/-*^ mouse lungs (Fig 1B–1D). We also instilled live PA and found reduced airway KC abundance and neutrophil recruitment in *Aoah*^*-/-*^ mice (S1 Fig). These data suggested that defective neutrophil recruitment may increase susceptibility to pulmonary infections in *Aoah*^*-/-*^ mice [7].

### In *Aoah*^*-/-*^ mice, both alveolar macrophages and epithelial cells have reduced innate responses to LPS *in vivo*

**Fig 2:**
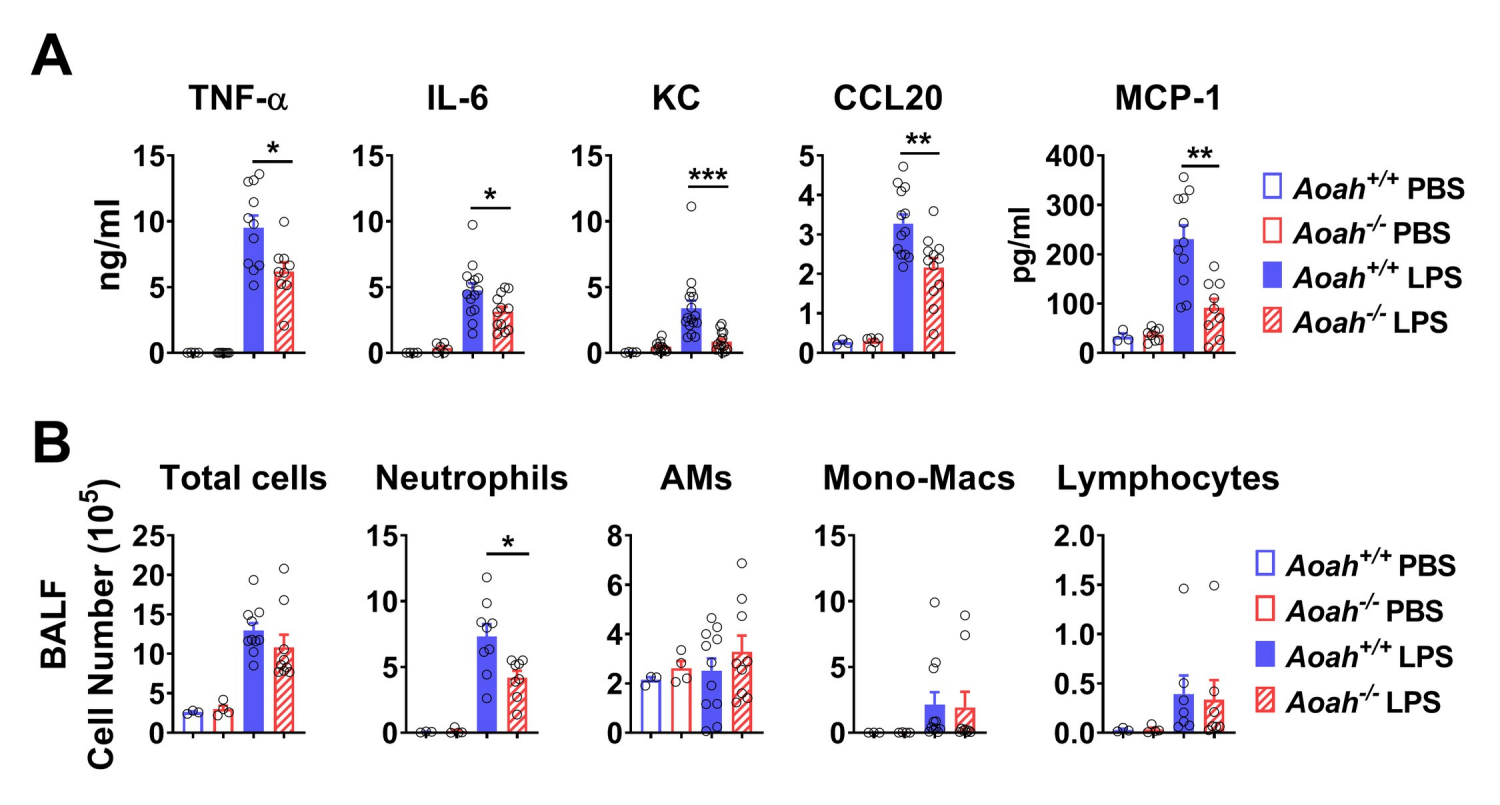
The innate immune responses to LPS of both AMs and alveolar epithelial cells are decreased in *Aoah*^*-/-*^ mice *in vivo*. (A) *Aoah*^*+/+*^ and *Aoah*^*−/−*^ mice were instilled with 10 μg LPS i.n. or PBS i.n. as controls. Five h later, the concentrations of inflammatory cytokines and chemokines in BALF were determined using ELISA. (B) Cells in BALF were counted and analyzed using FACS. (A, B) Data were combined from 4 experiments. n = 7–16. Mann-Whitney test was used. *, P < 0.05; **, P < 0.01; ***, P < 0.001.

We next tested whether *Aoah*^*-/-*^ mice had reduced innate responses to inhaled LPS. Five hours after LPS was instilled i.n., the bronchoalveolar lavage fluid (BALF) from *Aoah*^*-/-*^ mice contained less TNF-α, IL-6, KC, CCL20 and MCP-1 (CCL-2) than did BALF from *Aoah*^*+/+*^ mice (Fig 2A). In line with previous results [24,33–36], we found that TNF-α is mainly produced by AMs; IL-6 and KC by both AMs and alveolar epithelial cells (AECs); and MCP-1, MIP-2, GM-CSF and CCL20 mainly by AECs (S2 Fig). Thus, both AMs and AECs are hypo-responsive to LPS stimulation in *Aoah*^*-/-*^ mice. LPS instillation also recruited fewer neutrophils to the airways and lungs of *Aoah*^*-/-*^ mice (Figs 2B and S3).

### *Aoah*^*-/-*^ alveolar macrophages have reduced innate responses to LPS *in vitro*

**Fig 3:**
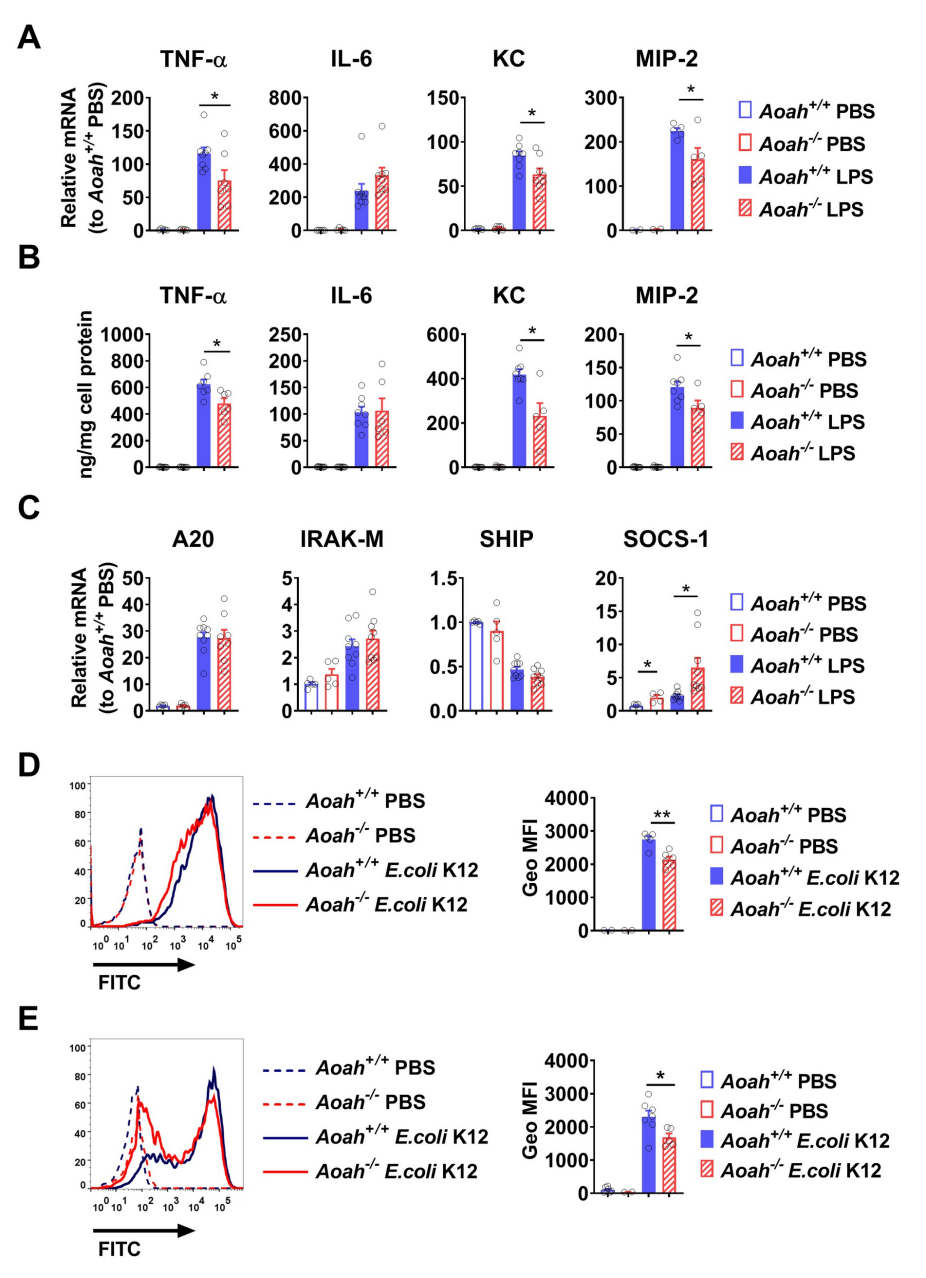
*Aoah*^*-/-*^ alveolar macrophages have reduced innate responses to LPS *in vitro*. (A) AMs in BALF were allowed to adhere to plastic plates. Then they were treated with PBS or 10 ng/ml LPS *in vitro* for 2 h. The mRNAs were measured using quantitative real-time PCR. Data were combined from 2 or 3 experiments, n = 5–9. (B) AMs were treated with PBS or 10 ng/ml LPS *in vitro* for 6 h and the released cytokines or chemokines were measured in the culture media. Data were combined from 2 or 3 experiments, n = 5–8. (C) AMs were isolated and treated with PBS or 10 ng/ml LPS *in vitro* for 2 h. The mRNAs were measured using quantitative real-time PCR. The expression levels in the *Aoah*^*+/+*^ PBS group were set to 1 and the relative expression levels of genes in the other groups were calculated. Data were combined from 2 or 3 experiments, n = 5–9. (D) AMs were cultured in RPMI 1640 containing 10% mouse serum in low-adherence plates. FITC-*E*. *coli* K12 at a ratio of 50 bacteria/cell or PBS was added. After 2 h incubation, cells were collected. After the extracellular FITC was quenched by trypan blue, the geometric mean fluorescence intensity (Geo MFI) of FITC was measured in AMs using FACS. Data were combined from 2 experiments, n = 5 or 6. (E) Mice were instilled i.n. with 2 × 10^7^ FITC-*E*. *coli* K12. Two h later, BALF cells were stained with anti-CD11c Ab and subjected to FACS analysis. AMs were gated as CD11c^hi^ cells and the Geo MFI of FITC was measured. Data were combined from 2 experiments, n = 5–7. (A-E) Mann-Whitney test was used. *, P < 0.05; **, P < 0.01.

AMs are known to play a critical role in pulmonary host defense [37–39]. To confirm that *Aoah*^*-/-*^ AMs had reduced responsiveness, we isolated *Aoah*^*+/+*^ and *Aoah*^*-/-*^ AMs and stimulated them with LPS *in vitro*. LPS induced less TNF-α, KC, and MIP-2 mRNA production (Fig 3A) and protein secretion (Fig 3B) in *Aoah*^*-/-*^ AMs than in *Aoah*^*+/+*^ AMs. We also measured the mRNA levels of 4 negative regulators of the TLR signaling pathway. The expression of suppressor of cytokine signalling-1 (SOCS-1) was elevated in *Aoah*^*-/-*^ AMs and robustly induced by LPS stimulation *in vitro*, which may contribute to reduced TNF-α, KC and MIP-2 expression [40,41] (Fig 3C). The expression of other negative regulators, including A20, IRAK-M or SHIP, was similar in *Aoah*^*+/+*^ and *Aoah*^*-/-*^ AMs both before and after LPS stimulation (Fig 3C). As AMs are slowly replaced by recruited monocytes [42,43], we tested whether monocyte progenitors in bone marrow and blood monocytes in *Aoah*^*-/-*^mice were already tolerant. We found that upon LPS treatment, similar proportions of *Aoah*^*-/-*^ and *Aoah*^*+/+*^ monocytes were stimulated to produce TNF-α and IL-6, suggesting that *Aoah*^*-/-*^ monocytes were not tolerant and thus that the lung microenvironment may account for AM tolerance (S4 Fig). The ability of *Aoah*^*-/-*^ AMs to phagocytose *E*. *coli* was reduced compared with that of *Aoah*^*+/+*^ AMs *in vitro* and *in vivo* (Fig 3D and 3E). Thus, *Aoah*^*-/-*^ AMs are tolerant: they are hypo-responsive to LPS stimulation and less phagocytic, properties that may contribute to increased susceptibility to pulmonary infections.

### *Aoah*^*-/-*^ alveolar macrophages have metabolic changes characteristic of tolerant monocytes

**Fig 4:**
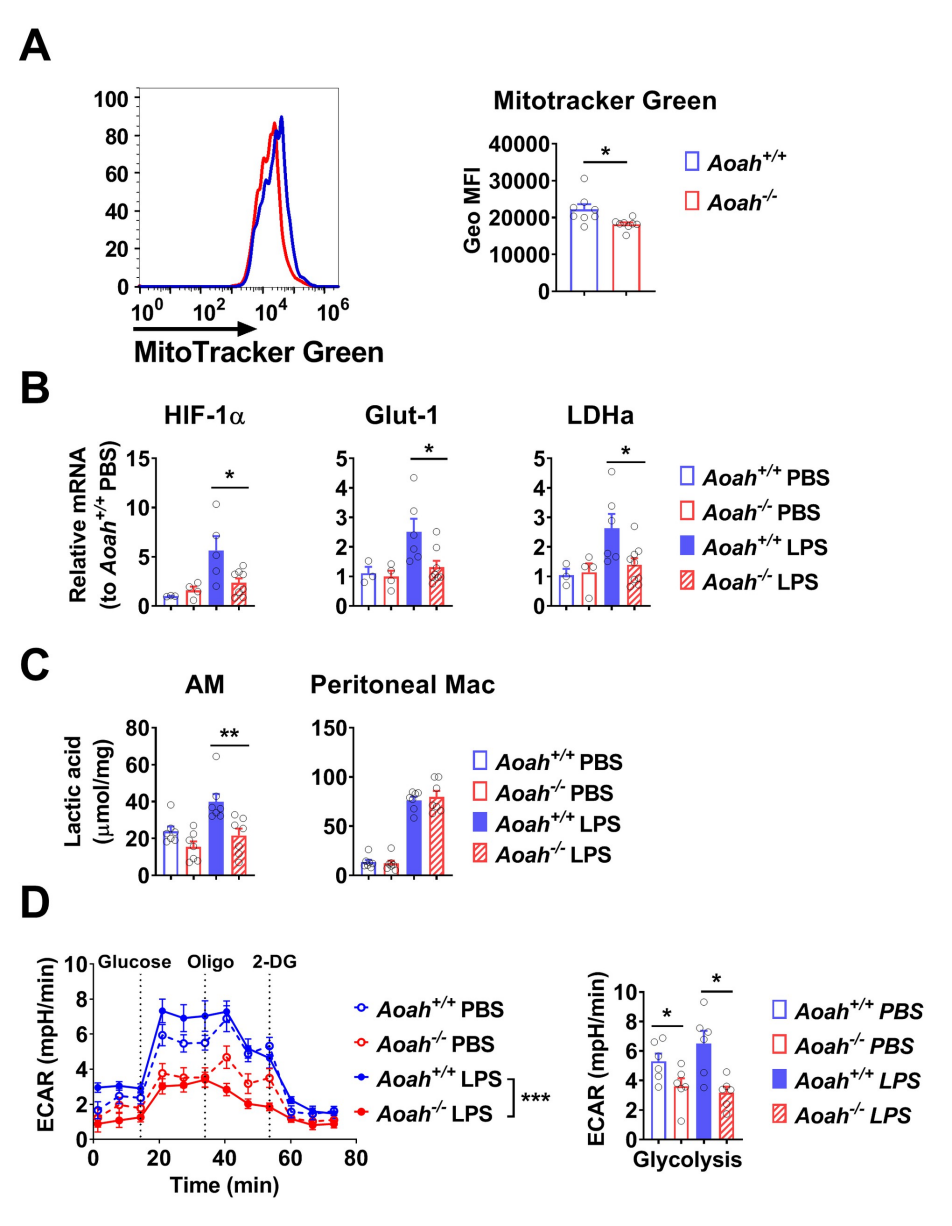
*Aoah*^*-/-*^ alveolar macrophages have metabolic changes characteristic of tolerant monocytes. (A) AMs were stained with Mitotracker Green and analyzed using flow cytometry. Histogram overlay of representative *Aoah*^*+/+*^ and *Aoah*^*-/-*^ AMs (left panel). Geometric mean florescence intensity (Geo MFI) of Mitotracker Green on AMs was measured (right panel). Data were combined from 3 experiments, n = 8. (B) AMs were treated with PBS or 10 ng/ml LPS *in vitro* for 2 h. mRNA was measured using qPCR. The unstimulated expression levels of *Aoah*^*+/+*^ (PBS) were set to 1 and the relative expression levels of genes in other groups were calculated. Data were combined from 2 experiments, n = 3–9. (C) AMs were cultured in RPMI 1640 containing 0.5% FBS and treated with PBS or 10 ng/ml LPS for 24 h. Lactate in the culture media was measured. Data were combined from 2 experiments, n = 7. (D) AMs were cultured in RPMI containing 5% FBS and treated with PBS or 10 ng/ml LPS for 24 h. ECARs were accessed using the Seahorse technology. Glycolysis was measured after the addition of 10 mM glucose. Data were combined from 2 experiments, n = 5–6. (A-D) Mann-Whitney test and Two-way ANOVA test (D, ECAR) were used. *, P < 0.05; **, P < 0.01; ***, P < 0.001.

Cheng et al. studied monocytes from septic patients and found that the immunotolerant monocytes had reduced OXPHOS activity and produced less lactate upon *in vitro* LPS stimulation [44]. Similarly, we found that *Aoah*^*-/-*^ AMs had slightly reduced mitochondrial mass (Fig 4A). Unlike peritoneal macrophages or bone marrow derived macrophages, AMs only slightly increase glycolysis upon LPS stimulation, yet when AMs were explanted and cultured *in vitro*, they increased the expression of some glycolytic pathway genes [45–47]. When AMs were explanted and stimulated with LPS, the expression of HIF-1α and its target glycolytic genes Glut-1 and LDHa was not induced in *Aoah*^*-/-*^ AMs (Fig 4B). Accordingly, *Aoah*^*-/-*^ AMs produced less lactate than did *Aoah*^*+/+*^ AMs, while *Aoah*^*-/-*^ and *Aoah*^*+/+*^peritoneal macrophages produced similar amounts of lactate (Fig 4C). When we measured extracellular acidification rate (ECAR) of AMs, we found that after glucose was added, *Aoah*^*-/-*^ AMs had reduced ECAR compared with *Aoah*^*+/+*^ AMs, with or without LPS stimulation (Fig 4D). These data suggest that, like tolerant monocytes in septic patients [44], *Aoah*^*-/-*^ AMs have reduced mitochondrial function; they do not increase glycolysis upon LPS stimulation *in vitro*, in keeping with their reduced cytokine or chemokine secretion (Fig 3).

### *Aoah*^*-/-*^ AMs have increased MHC II and costimulatory molecule expression

**Fig 5:**
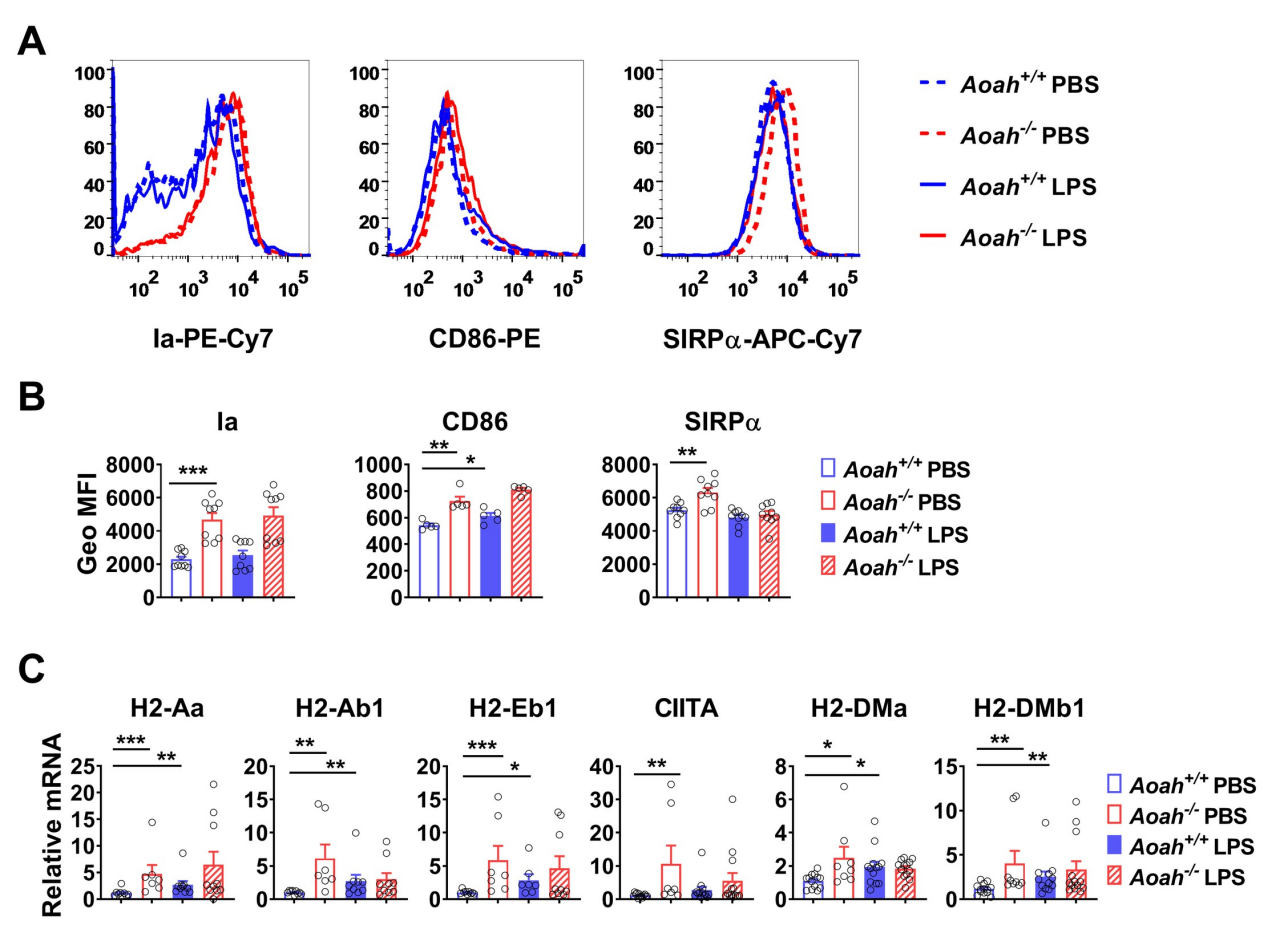
*Aoah*^*-/-*^ AMs express more MHC class II and costimulatory molecules. (A and B) *Aoah*^*+/+*^ and *Aoah*^*-/-*^ AMs were collected, treated with PBS or 1 ng/ml LPS for 6 h before the cell-surface expression of Ia, CD86 and SIRP-α was measured using FACS (A). The Geo MFI of Ia, CD86 and SIRPα was measured (B). (C) *Aoah*^*+/+*^ and *Aoah*^*-/-*^ AMs were collected, treated with PBS or 1 ng/ml LPS for 2 h before MHC II, CIITA and DM mRNA was measured. (A-C) Data were combined from 2 experiments, n = 5–9. Mann-Whitney test was used. *, P < 0.05; **, P < 0.01; ***, P < 0.001.

As explanted *Aoah*^*-/-*^ AMs showed characteristics of endotoxin tolerance, we asked whether they had been activated *in vivo*. Upon MAMP stimulation, macrophages increase MHC II and co-stimulatory molecule expression [48]. We found that *Aoah*^*-/-*^ AMs had increased cell surface expression of MHC II molecule Ia (H2-A) and costimulatory molecule CD86 (Fig 5A and 5B). *Aoah*^*-/-*^ AMs also had increased expression of SIRP-α (Fig 5A and5B), a negative regulator of tyrosine kinase-coupled signaling as well as phagocytosis [49,50], in keeping with their decreased phagocytic activity (Fig 3E and 3F). In addition, we found that *Aoah*^*-/-*^ AMs had increased MHC II (H2-Aa, H2-Ab1, H2-Eb1), CIITA (Class II Major Histocompatibility Complex Transactivator) and DM (H2-DMa, H2-DMb1, which assists MHC II peptide loading) mRNA; LPS stimulation increased MHC II and DM mRNA in *Aoah*^*+/+*^ AMs (Fig 5C). Increased Ia and CD86 expression provides additional evidence that *Aoah*^*-/-*^ AMs may be exposed to endogenous MAMPs that induce innate tolerance and reduce pulmonary defenses.

### Circulating or gut-derived LPS inhibits pulmonary innate responses

**Fig 6:**
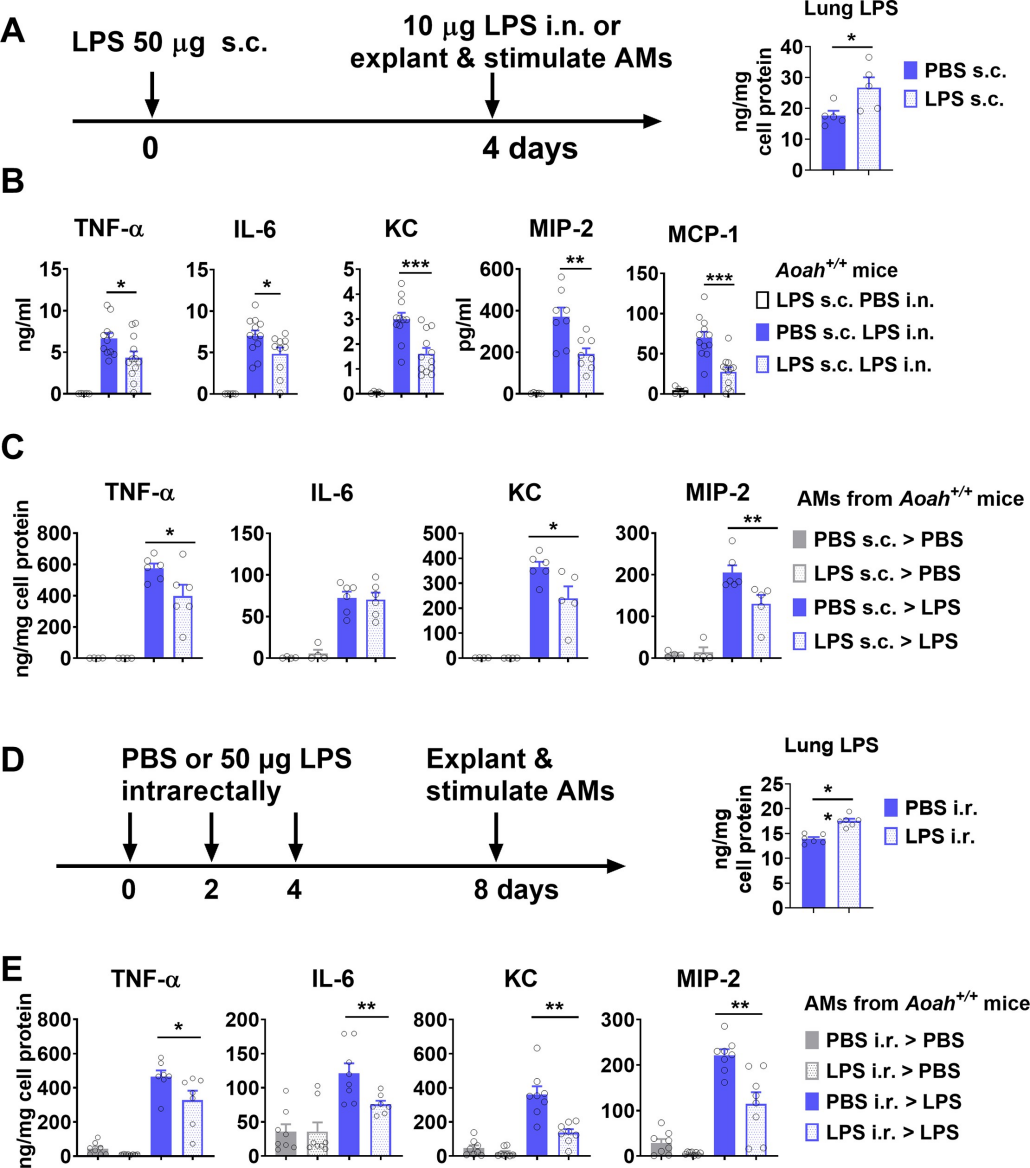
Circulating LPS inhibits pulmonary innate responses in *Aoah*^*+/+*^ mice. (A-C) *Aoah*^*+/+*^ mice were treated with 50 μg LPS s.c. (footpad injection). Four days later, in some experiments, lung LPS was measured, n = 5 (A). In other experiments, 10 μg LPS was instilled i.n. and 5 h later, BALF cytokine levels were measured using ELISA. Data were combined from 3 experiments. n = 8–12 (B). Four days later after LPS s.c. injection, *Aoah*^*+/+*^ mouse AMs were explanted and treated with PBS or 10 ng/ml LPS for 6 h *ex vivo*. The culture media were collected for cytokine and chemokine ELISA. Data were combined from 2 experiments. n = 5 or 6 (C). (D, E) PBS or 50 μg LPS was given intrarectally on day 0, 2 and 4 to *Aoah*^*+/+*^ mice. On day 8, in some experiments, lung LPS was measured, n = 6 (D). In other experiments, AMs were explanted and stimulated with 10 ng/ml LPS. After 6 h treatment, TNF-α, IL-6, KC and MIP-2 were measured in culture media using ELISA (E). Data were combined from 2 experiments. n = 7 or 8. (A–E) The Mann-Whitney test was used. *, P < 0.05; **, P < 0.01; ***, P < 0.001.

As we have found that LPS derived from gut commensal Gram-negative bacteria may desensitize lung epithelial cells and reduce their responses to house dust mite allergen [31], we asked whether the reduced AM response to LPS or *Pseudomonas aeruginosa* was also induced by gut-derived LPS. First, we tested whether or not circulating LPS can tolerize AMs. After s.c. injection, LPS drains slowly via lymphatics to reach the bloodstream [22]. We injected LPS into a mouse footpad and 4 days later we instilled LPS i.n., waited 5 h, and measured inflammatory cytokines and chemokines in the BALF (Fig 6A). Subcutaneous LPS injection increased lung LPS levels (Fig 6A). Mice pretreated with LPS had reduced innate responses to inhaled LPS (Fig 6B). In addition, after we explanted AMs and stimulated them with LPS, we found that TNF-α, KC and MIP-2 secretion was significantly reduced in mice that had received LPS s.c., providing evidence that circulating LPS is able to tolerize AMs (Fig 6C). To study whether gut-derived LPS can tolerize AMs, we gave 50 μg LPS intrarectally to *Aoah*^*+/+*^ mice on days 0, 2, 4, and on day 8, we explanted AMs, and stimulated them with LPS *in vitro* (Fig 6D). Intrarectally administered LPS increased lung LPS levels (Fig 6D). AMs from LPS-treated mice had significantly lower TNF-α, IL-6, KC and MIP-2 secretion (Fig 6E). These results suggest that gut-derived LPS can get to the lung via circulation and tolerizes AMs.

### Intestinal LPS regulates pulmonary innate responses in a TLR4-dependent manner

**Fig 7:**
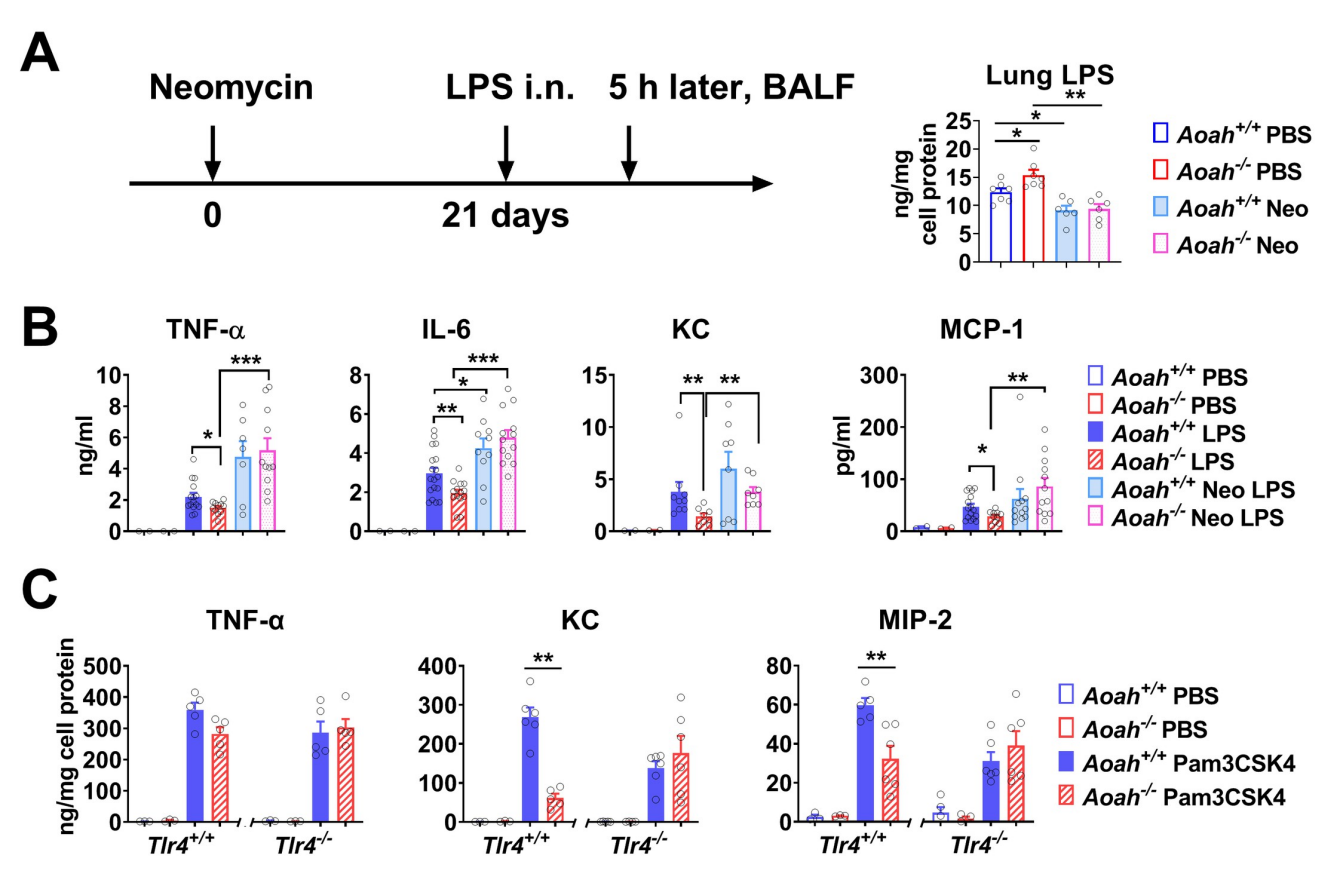
Intestinal LPS regulates pulmonary innate responses; TLR4 signaling is required for AM tolerance. (A) *Aoah*^*+/+*^ and *Aoah*^*−/−*^ mice were co-housed and treated with neomycin (1 g/L) in drinking water for 3 weeks and the lung LPS was measured. n = 6 or 7. (B) Five h after 10 μg LPS was instilled i.n., BALF cytokine levels were measured using ELISA. Data were combined from 2 or 3 experiments. n = 7–15. (C) AMs were isolated from naive *Aoah*^*+/+*^, *Aoah*^*-/-*^, *Aoah*^*+/+*^*TLR4*^*-/-*^, and *Aoah*^*-/-*^*Tlr4*^*-/-*^ mice. Then they were treated with PBS or 10 ng/ml Pam3CSK4 for 6 h. TNF-α, KC and MIP-2 were measured in the culture medium. Data were combined from two experiments. n = 5 or 6. (A–C) Mann-Whitney test was used. *, P < 0.05; **, P < 0.01; ***, P < 0.001.

We found previously that *Aoah*^*-/-*^ mouse feces had more bioactive LPS than did that of *Aoah*^*+/+*^ mice and that antibiotic treatment reduced the levels of bioactive LPS in *Aoah*^*-/-*^ mouse feces and blood [31]. To find out whether intestinal commensal LPS also modulates AM responsiveness, we added neomycin to drinking water to deplete commensal aerobic Gram-negative bacteria (Fig 7A). Neomycin is poorly absorbed from the gastrointestinal tract. Neomycin treatment reduced lung LPS levels (Fig 7A), significantly increased *in vivo* innate responses to LPS in the lungs, and diminished the difference between *Aoah*^*+/+*^ and *Aoah*^*-/-*^ mouse lung responses (Fig 7B), suggesting that exposure to gut commensal LPS desensitizes AM responses. These data provide further evidence that LPS derived from gut commensals may translocate into the bloodstream, travel to the lung, and decrease innate responses to LPS in AMs.

As we had evidence that gut-derived LPS tolerizes AMs, we hypothesized that the hypo-responsiveness of *Aoah*^*-/-*^ AMs relies upon TLR4 sensing of LPS and that when the LPS sensor TLR4 is missing, *Aoah*^*-/-*^ and *Aoah*^*+/+*^ AMs have similar responsiveness. We treated AMs from naïve *Aoah*^*+/+*^, *Aoah*^*-/-*^, *Tlr4*^*-/-*^ and *Aoah*^*-/-*^*Tlr4*^*-/-*^ mice with a TLR2 ligand (Pam3CSK4) *in vitro*. When TLR4 was present, *Aoah*^*-/-*^ AMs produced less KC and MIP-2 than did *Aoah* ^*+/+*^ AMs, providing evidence that *Aoah*^*-/-*^ AMs were also hypo-responsive to other TLR agonists in addition to LPS (cross-tolerance or hetero-tolerance [51]) (Fig 7C). When TLR4 was lacking, the difference between innate responses in *Aoah*^*+/+*^ and *Aoah*^*-/-*^ AMs diminished, suggesting that TLR4 sensing is required to produce *Aoah*^*-/-*^ AM tolerance (Fig 7C).

## Discussion

Discovered in the 1980s, AOAH is a highly conserved and unique host lipase that deacylates LPS [19]. By removing the secondary fatty acyl chains from lipid A, AOAH converts stimulatory hexaacyl LPS to antagonistic tetraacyl LPS. We found previously that AOAH is required to prevent prolonged endotoxin tolerance in the peritoneal macrophages of LPS-exposed mice [12,26]. In this study, we found that AOAH also may sensitize alveolar macrophages (AMs) by decreasing the abundance of bioactive LPS in the gut.

*Aoah*^*-/-*^ mice were more susceptible to *Pseudomonas aeruginosa* (PA)-induced pulmonary infection. Upon inhaling *P*. *aeruginosa*, *Aoah*^*-/-*^ mice secreted less KC and recruited fewer neutrophils to their lungs than did *Aoah*^*+/+*^ mice. Cytokines produced by both alveolar macrophages and epithelial cells were reduced in responses to intranasally instilled LPS *Aoah*^*-/-*^ mice. When we stimulated AMs *in vitro*, *Aoah*^*-/-*^ AMs were hypo-responsive and less phagocytic than were *Aoah*^*+/+*^ AMs, confirming that the *Aoah*^*-/-*^ AMs were tolerant. Interestingly, *Aoah*^*-/-*^ AMs had reduced mitochondrial mass and could not increase glycolysis upon LPS re-stimulation, properties that resemble those reported for tolerant monocytes from septic patients [44]. *Aoah*^*-/-*^ AMs also expressed high levels of MHC II and co-stimulatory molecules, suggesting that they were stimulated *in situ*. We further showed that circulating LPS coming from a distal subcutaneous injection site or intrarectally administered LPS could tolerize AMs, while antibiotic treatment that reduced colonic commensal Gram-negative bacteria prevented AM tolerance in *Aoah*^*-/-*^ mice. Importantly, TLR4 sensing was required for tolerance to be maintained in *Aoah*^*-/-*^ AMs. We conclude that by deacylating commensal LPS in the gut, AOAH prevents AM tolerance and therefore increases resistance to pulmonary infections.

Many previous studies have found that the intestinal microbiota shapes lung immunity [28–30]. Gut microbiota are required for pulmonary defense against bacterial or viral infections [52–55]. On the other hand, dysbiosis or excessive translocation of gut commensal bacteria or their components into the circulation may reduce host defense in the lungs [29,56]. Mason et al. found that injection of LPS into the portal vein led to reduction in *P*. *aeruginosa* clearance after aerosol challenge, with reduced neutrophil recruitment, decreased AM phagocytic activity and diminished TNF-α production in the lung, findings suggesting that excessive gut-derived LPS can compromise lung defense [57]. In this study, we found that when an elevated amount of bioactive LPS was present in the intestine of *Aoah*^*-/-*^ mice, AMs become tolerized. Previously, we found that *Aoah*^*-/-*^ mouse lung epithelial cells were also desensitized by gut-derived LPS [31]. As pulmonary epithelial cells are essential for shaping innate immunity in the lung [38,58–60], desensitization of both epithelial cells and AMs may increase susceptibility to pulmonary infections additively or synergistically. Our data suggest that stimulation of TLR4 by gut-derived LPS is required for *Aoah*^*-/-*^ AM tolerance. As both AMs and AECs express TLR4, it is not clear whether direct stimulation of TLR4 on AMs induces tolerance or epithelial desensitization leads to AM tolerance. How AECs shape the innate responsiveness of AMs in *Aoah*^*-/-*^ mice will be studied in the future.

We found that feces from *Aoah*^*-/-*^ mice contained more bioactive LPS than did those from co-housed wildtype mice [31]. AOAH mRNA is expressed in the small and large intestines, mainly in macrophages and dendritic cells, both of which can take up Gram-negative bacteria or LPS and deacylate LPS [31,61–63]. AOAH may also be secreted and taken up by non-AOAH producing cells and deacylate LPS [64,65]. In addition, extracellular AOAH may act on LPS in the gut lumen [66]. Intestinal LPS that reaches the liver via portal vein may be further deacylated by AOAH produced by Kupffer cells, NK cells and dendritic cells [23,25,67]. How and where intestinal LPS is deacylated by AOAH awaits further investigation.

The tolerant *Aoah*^*-/-*^ AMs had distinctive features. First, unlike macrophages in other organs, AMs were not tolerized after intravenous LPS injection [68–71], and in unpublished studies we found that intranasal instillation of LPS partially primed AMs. Acute exposure to intravenous or inhaled LPS may reprogram AMs differently from chronic low-level LPS exposure in *Aoah*^*-/-*^ mice. Indeed, we found that subcutaneously-injected LPS also induced AM tolerance instead of priming the cells. Second, tolerant *Aoah*^*-/-*^ AMs had reduced phagocytic activity; other studies have found that tolerant macrophages are more phagocytic [72,73]. Third, upon PAMP stimulation, tolerant *Aoah*^*-/-*^ AMs secreted less KC, which leads to reduced neutrophil recruitment, while Ariga et al., found that endotoxin tolerance promotes neutrophil recruitment to infected sites [74]. The discrepancy may be due to LPS exposure dose and time, the tissue microenvironment, and macrophage origins (tissue resident or monocyte-derived).

Previously, we found that after LPS i.p. injection, tolerant *Aoah*^*-/-*^ peritoneal macrophages had increased expression of a TLR signaling negative regulator, IRAK-M [12,26], while A20, another negative regulator, was induced in *Aoah*^*-/-*^ mouse lung epithelial cells [31]. In this study we found that SOCS-1 was upregulated in *Aoah*^*-/-*^ AMs before and after LPS stimulation, while IRAK-M, A20 and SHIP expression was unchanged. SOCS-1 negatively regulates TLR signaling and is responsible for endotoxin tolerance [40,41,75]. Notably, SOCS-1 can be secreted by AMs in exosomes and inhibits STAT1 activation in lung epithelial cells [76]. SOCS-1 also limits STAT3/HIF-1α axis activation and reduces glycolysis in peritoneal cells [77], in line with our findings that *Aoah*^*-/-*^ AMs have increased SOCS-1 expression and reduced glycolysis upon LPS re-stimulation.

Tolerant *Aoah*^*-/-*^ AMs had higher surface expression of MHC II and CD86 molecules. Long-lived AMs reside in alveoli and maintain their abundance by self-renewal [38]. In naïve mice, a small number of monocytes contribute to the AM pool, while during lung inflammation and injury, monocytes are recruited to the alveoli [38,43]. When *Aoah*^*-/-*^ lungs are constantly exposed to low levels of gut-derived LPS, resident AMs may increase their MHC II molecule and co-stimulatory molecule expression. In contrast, 14 days after LPS i.p. injection, tolerant *Aoah*^*-/-*^ peritoneal macrophages expressed low levels of CD86 [26]. The discrepancy may be that 14 days after LPS i.p. injection, approximately 75% of peritoneal macrophages are derived from recruited monocytes [78] and monocyte-derived macrophages may maintain low levels of CD86 expression when bioactive LPS persists in *Aoah*^*-/-*^ mouse peritoneum.

As AMs are at the first line of defense against airborne pathogens and can be obtained for study with minimal disturbance, we focused on these cells and did not study other cells that may participate in pulmonary host defense. It should be of interest to find out if lung interstitial macrophages, such as recently-discovered immunoregulatory CD169^+^ nerve- and airway-associated macrophages [79] or Lyve1^lo^MHCII^hi^CX3CR1^hi^ and Lyve1^hi^MHCII^lo^CX3CR1^lo^ macrophages [80] also develop persistent tolerance in *Aoah*^*-/-*^ mouse lungs. In addition, how altered AM metabolism contributes to immune responsiveness is also fertile ground for more detailed investigation.

In a previous study we found that AOAH promotes recovery from acute lung injury by inactivating inhaled LPS or the LPS produced by Gram-negative bacteria that enter the lung [24]. We also found that by deacylating gut commensal LPS, AOAH sensitizes pulmonary epithelial cells for allergen stimulation [31]. Here we provide evidence that the enzyme, by deacylating LPS in the intestine, also prepares alveolar macrophages to carry out pulmonary antibacterial defense. This highly conserved enzyme thus modulates pulmonary mucosal immunity by degrading LPS that reaches the lungs from both endogenous and exogenous sources.

## Materials and methods

### Ethics statement

All mice were housed under specific pathogen-free conditions in Fudan University, the Department of Laboratory Animal Science, and studied using protocols approved by the Institutional Animal Care and Use Committee (IACUC) of Fudan University (approved animal protocol number 20170816–002). All protocols adhered to the guide for the Care and Use of Laboratory Animals.

### Mice

C57BL/6J *Aoah*^*+/+*^, *Aoah*^*−/−*^ and *Tlr4*^*−/−*^*Aoah*^*−/−*^ mice were obtained from the laboratory of Dr. Robert Munford at the National Institutes of Health, Bethesda, MD, USA. The generation of *Aoah*^*−/−*^ mice has been described previously (Lu et al., 2003). The mutated *Aoah* gene had been backcrossed to C57BL/6J mice for at least 10 generations. *Tlr4*^*−/−*^*Aoah*^*−/−*^ mice were produced by crossing *Aoah*^*−/−*^ and *Tlr4*^*−/−*^ mice. *Aoah*^*+/+*^ and *Aoah*^*-/-*^ mice were cohoused for at least 3 weeks before the start and throughout the experiments. We found previously that co-housed *Aoah*^*+/+*^ and *Aoah*^*-/-*^ mice had similar microbiota and that *Aoah*^*-/-*^ mouse feces had more bioactive LPS than did *Aoah*^*+/+*^ mouse feces [31].

### Reagents

Anti-mouse antibodies used for flow cytometry were anti-CD45-BV785 (Clone 30-F11, BioLegend), anti-CD11b-FITC (Clone M1/70, BD), anti-CD11c-V450 (Clone N418, eBioscience), anti-Ly6G-FITC (Clone 1A8, BD), anti-SiglecF-PE (Clone E50-2440, BioLegend), anti-MHC II-PE-Cy7 (Clone M5/114.15.2, BD bioscience), anti-Ly6C-APC-Cy7 (Clone HK1.4, BD bioscience), anti-CD64-AF647 (Clone X54-5/7.1, eBioscience), anti-IL-6-PE (Clone MP5-20F3, BD bioscience) and anti-TNF-α-APC (Clone MP6-XT22, BD bioscience). Mouse IL-6, TNF-α, and MCP-1 ELISA kits were from BD; KC, CCL20 and MIP-2 kits were from R&D system. Lactic Acid LD test kit was obtained from Nanjing Jiancheng Bioengineering Institute.

### *P*. *aeruginosa* culture and infection

The prototypic strain of *Pseudomonas aeruginosa* PAO1 was inoculated and cultured for 18 h in LB Broth (Difco, BD Diagnostics) at 37°C with constant shaking. The bacteria were then centrifuged and the pellet was re-suspended in PBS. The bacterial suspension was adjusted to OD_650_ = 0.5, which contained about 8 × 10^8^ colony forming units (CFU)/ml. The bacterial suspension was diluted and spread on LB plates to confirm the bacterial concentration. After *Aoah*^*+/+*^ and *Aoah*^*-/-*^ mice were anesthetized with 0.5% pentobarbital sodium (50 μg/g body weight) i.p., about 3 × 10^6^ CFU live PAO1 or 1 × 10^7^ heat-killed (boiled for 15 minutes) PAO1 in 40 μl PBS were instilled intranasally. Before and after infection, mouse body weight was measured daily for 7 days. In some experiments, 2 days after infection, mice were euthanized and their blood and lungs were collected. The lungs were aseptically dissected and homogenized in 1 ml sterile PBS. The blood and tissue homogenates were diluted and spread on LB plates. The plates were incubated at 37°C for 18 h, and CFUs were counted to determine lung bacterial load. In other experiments, bronchoalveolar lavage (BALF) was collected for immune cell analysis and cytokine concentration measurement 5 h after bacterial instillation.

### Bronchoalveolar lavage (BALF) analysis

BALF was obtained as described in a previous study [24]. Briefly, mice were anesthetized and exsanguinated by cutting the inferior vena cava. Bronchoalveolar lavage was performed using 1 ml of EDTA-containing PBS for 5 times. The BALF collected from one mouse was combined and centrifuged at 1500 rpm for 5 min at 4°C. The supernatant was used for cytokine or chemokine ELISA and the cell pellet was re-suspended in PBS. The cells were counted using Cellometer (Nexcelom) and the cells were stained and analyzed using FACS.

### Isolation and culture of AMs

After BALF was collected and centrifuged, the cell pellet was resuspended in complete RPMI medium, which contained 10% fetal bovine serum (Hyclone), 2 mM glutamine, 100 U/ml penicillin, and 0.1 mg/ml streptomycin (Life Technologies). BALF cells were incubated at 37°C for 4 h and AMs were allowed to adhere to plastic plates. The floating cells were then washed away and AMs were treated with PBS, 10 ng/ml LPS O111 (Sigma) or 10 ng/ml Pam3CSK4 (Invivogen, TLR1/2 agonist) for 6 h. The culture media were collected for cytokine or chemokine ELISA and the cells were lysed for protein measurement. To measure cytokine or chemokine mRNA, AMs were stimulated for 2 h and the cells were lysed for qPCR. In some experiments, *Aoah*^*+/+*^ mice were injected with 50 μg LPS in the left footpad. After 4 days, the mice were instilled with 10 μg LPS i.n. and their BALF was collected 5 h later for ELISA; or their BALF was harvested and AMs were explanted for *in vitro* LPS stimulation.

### Lung digestion and single cell preparation

To measure immune cells in the lung, the lungs were perfused, excised, then cut into 1 mm^3^ pieces and incubated at 37°C for 1 h while shaking in digestion buffer, which contained RPMI 1640 (Gibco), 1 mg/ml collagenase IV (Sigma) and 10 U/ml DNase I (Sigma). The digested lung tissues were filtered through a 70 mm cell strainer. Red blood cells were then lysed using ACK lysis buffer (eBioscience). Cells were stained with antibodies and subjected to flow cytometric analysis or magnetic activated cell sorting.

### Flow cytometry

BALF or lung single cell suspension was obtained after PBS, LPS or heated-*P*. *aeruginosa* i.n. instillation. Lung cells were collected by centrifugation and then incubated with Fc blocking antibody (purified anti-mouse CD16/32, BioLegend) on ice for 15 min. After the cells were stained with fluorescence-conjugated antibodies for 30 min on ice, the cells were washed and subjected to FACS (BD, FACSCelesta). The FACS data were analyzed using Flow Jo software (TreeStar,Inc). All antibodies used for flow cytometry were anti-mouse antigens.

### Quantitative real-time PCR (qPCR)

RNA from AMs was isolated using TRNzol Universal Reagent (Tiangen) and reversely transcribed (Tiangen). The primers used for qPCR were listed in S1 Table. Actin was used as an internal control and the relative gene expression was calculated using the ΔΔCt quantification method.

### Phagocytosis analysis

Alveolar macrophages were isolated from BALF and plated at 3 × 10^5^ macrophages/well in a low adherent 96-well tissue culture plate. FITC-labeled *E*. *coli* bioparticles (Vybrant Phagocytosis Assay; Invitrogen) were added to each well at a ratio of 50 bacteria/cell in RPMI medium with 10% fresh mouse serum, and the plates were incubated for 2 h at 37°C with 80 rpm shaking. AMs were then harvested, trypan blue was added to each well to quench the fluorescence of extracellular bacteria, and intracellular bacteria fluorescence was measured using FACS. Geometric mean florescence intensity (Geo MFI) of FITC was calculated. To measure phagocytic activity of AMs *in vivo*, mice were instilled i.n. with 2 × 10^7^ FITC *E*. *coli* K12. Two h later, BALF cells were collected, stained with anti-CD11c-APC Ab and subjected to FACS analysis. AMs were gated as CD11c^hi^ cells and the Geo MFI of FITC was measured.

### Blood and bone marrow monocyte innate response

Blood and bone marrow were taken from naïve *Aoah*^*+/+*^ or *Aoah*^*-/-*^ mice. After red blood cells were lysed using ACK lysis buffer, the single cells of blood or bone marrow were cultured in RPMI 1640 containing 5% FBS in low-adherent plates, and then treated with 10 ng/ml LPS. Brefeldin A (5 μg/ml, BioLegend) was added simultaneously to block cytokine secretion. Six h later, blood and bone marrow cells were washed, intracellular TNF-α, IL-6 and cell surface CD11b and Ly6C were stained before FACS analysis.

### Magnetic activated cell sorting (MACS)

To identify the source of cytokines or chemokines, AMs and Alveolar epithelial cells (AECs) were obtained from BALF or lungs respectively. AECs (CD45^-^ CD326^+^) were sorted using anti-CD45 and anti-CD326 antibody-conjugated magnetic beads (Miltenyi Biotec) according to the manufacturer’s instructions. The purity of CD45^-^ CD326^+^ cells was above 90% by flow cytometric analysis.

### ECAR analysis

AMs were plated at 3 × 10^4^ macrophages/well in a XF-96 plate and cultured in RPMI medium containing 5% FBS for 24 h at 37°C. The extracellular acidification rate (ECAR) was measured in a Seahorse XF extracellular flux analyzer (Agilent Technologies) according to the manufacturer’s instructions. Glucose (10 mM, Sigma), oligomycin (1 μM, Sigma) and 2-deoxyglucose (2-DG, 50 μM, Sigma) were used. Data were analyzed using Wave Desktop software version 2.6 (Agilent Technologies).

### Lactic acid measurement

Lactic acid produced by AMs was measured using Lactic Acid LD test kit (Nanjing Jiancheng Bioengineering Institute). After AMs were treated with PBS or LPS in RPMI medium containing 0.5% FBS for 24 h, culture media were collected, added to LDH (lactate dehydrogenase) working reagent with substrate and mixed well. The reaction was performed at 37°C for 10 min and the plates were read at 530 nm (Tecan). Lactic acid standards were used to generate standard curve for quantitation.

### Antibiotic treatment

To deplete intestinal commensal Gram-negative bacteria, mice were fed 1 g/L neomycin sulfate (Sigma) in their drinking water for at least 3 weeks before the mice were instilled 10 μg LPS intranasally.

### LPS quantification in lungs

Mouse lungs were dissected and homogenized in endotoxin-free PBS. After centrifugation, the supernatants were collected for TLR4-stimulating activity using a cell-based colorimetric endotoxin detection kit (HEK-Blue LPS Detection Kit2, Invivogen). In brief, diluted samples were added to human embryonic kidney (HEK-293) cells that express hTLR4 and an NF-κB–inducible secreted embryonic alkaline phosphatase reporter gene. After 18 h incubation, cell culture media were applied to QUANTI-Blue medium to measure alkaline phosphatase activity. Plates were read at a wavelength of 620 nm (Tecan).

### Statistical analysis

Data were presented as mean ± SEM. Difference between groups were analyzed using Mann-Whitney test. To compare kinetic difference, two-way ANOVA test was used. The statistical significance was set at P < 0.05. *, P < 0.05; **, P < 0.01; ***, P < 0.001.

## Supporting information

S1 Fig *Aoah*^*-/-*^ mice have reduced KC secretion and neutrophil recruitment after *Pseudomonas aeruginosa* infection. (DOCX)

S2 Fig After exposure to LPS, AMs and AECs express different cytokines/chemokines. (DOCX)

S3 Fig After LPS i.n. instillation, *Aoah*^*-/-*^ mouse lungs have reduced neutrophil proportion. (DOCX)

S4 Fig *Aoah*^*-/-*^ mouse blood or bone marrow monocytes are not tolerant. (DOCX)

S1 Table Primers used for qPCR. (DOCX)

## References

[1] Ballinger MN, Paine R 3rd, Serezani CH, Aronoff DM, Choi ES, Standiford TJ, et al. Role of granulocyte macrophage colony-stimulating factor during gram-negative lung infection with Pseudomonas aeruginosa. American journal of respiratory cell and molecular biology. 2006;34(6):766–74. doi: 10.1165/rcmb.2005-0246OC ; PubMed Central PMCID: PMC2644237. 16474098 PMC2644237

[2] Broug-Holub E, Toews GB, van Iwaarden JF, Strieter RM, Kunkel SL, Paine R 3rd, et al. Alveolar macrophages are required for protective pulmonary defenses in murine Klebsiella pneumonia: elimination of alveolar macrophages increases neutrophil recruitment but decreases bacterial clearance and survival. Infection and immunity. 1997;65(4):1139–46. doi: 10.1128/iai.65.4.1139-1146.1997 ; PubMed Central PMCID: PMC175109. 9119443 PMC175109

[3] Pittet LA, Quinton LJ, Yamamoto K, Robson BE, Ferrari JD, Algul H, et al. Earliest innate immune responses require macrophage RelA during pneumococcal pneumonia. American journal of respiratory cell and molecular biology. 2011;45(3):573–81. doi: 10.1165/rcmb.2010-0210OC ; PubMed Central PMCID: PMC3175578. 21216972 PMC3175578

[4] LeVine AM, Reed JA, Kurak KE, Cianciolo E, Whitsett JA. GM-CSF-deficient mice are susceptible to pulmonary group B streptococcal infection. The Journal of clinical investigation. 1999;103(4):563–9. doi: 10.1172/JCI5212 ; PubMed Central PMCID: PMC408099. 10021465 PMC408099

[5] Schneider C, Nobs SP, Heer AK, Kurrer M, Klinke G, van Rooijen N, et al. Alveolar macrophages are essential for protection from respiratory failure and associated morbidity following influenza virus infection. PLoS pathogens. 2014;10(4):e1004053. doi: 10.1371/journal.ppat.1004053 ; PubMed Central PMCID: PMC3974877. 24699679 PMC3974877

[6] Paine R 3rd, Preston AM, Wilcoxen S, Jin H, Siu BB, Morris SB, et al. Granulocyte-macrophage colony-stimulating factor in the innate immune response to Pneumocystis carinii pneumonia in mice. Journal of immunology. 2000;164(5):2602–9. doi: 10.4049/jimmunol.164.5.2602 . 10679099

[7] Didierlaurent A, Goulding J, Patel S, Snelgrove R, Low L, Bebien M, et al. Sustained desensitization to bacterial Toll-like receptor ligands after resolution of respiratory influenza infection. The Journal of experimental medicine. 2008;205(2):323–9. doi: 10.1084/jem.20070891 ; PubMed Central PMCID: PMC2271005. 18227219 PMC2271005

[8] Ziltener P, Reinheckel T, Oxenius A. Neutrophil and Alveolar Macrophage-Mediated Innate Immune Control of Legionella pneumophila Lung Infection via TNF and ROS. PLoS pathogens. 2016;12(4):e1005591. doi: 10.1371/journal.ppat.1005591 ; PubMed Central PMCID: PMC4841525. 27105352 PMC4841525

[9] Maus UA, Koay MA, Delbeck T, Mack M, Ermert M, Ermert L, et al. Role of resident alveolar macrophages in leukocyte traffic into the alveolar air space of intact mice. American journal of physiology Lung cellular and molecular physiology. 2002;282(6):L1245–52. doi: 10.1152/ajplung.00453.2001 . 12003780

[10] Kooguchi K, Hashimoto S, Kobayashi A, Kitamura Y, Kudoh I, Wiener-Kronish J, et al. Role of alveolar macrophages in initiation and regulation of inflammation in Pseudomonas aeruginosa pneumonia. Infection and immunity. 1998;66(7):3164–9. doi: 10.1128/IAI.66.7.3164-3169.1998 ; PubMed Central PMCID: PMC108328. 9632581 PMC108328

[11] Biswas SK, Lopez-Collazo E. Endotoxin tolerance: new mechanisms, molecules and clinical significance. Trends in immunology. 2009;30(10):475–87. doi: 10.1016/j.it.2009.07.009 . 19781994

[12] Lu M, Varley AW, Ohta S, Hardwick J, Munford RS. Host inactivation of bacterial lipopolysaccharide prevents prolonged tolerance following gram-negative bacterial infection. Cell host & microbe. 2008;4(3):293–302. doi: 10.1016/j.chom.2008.06.009 ; PubMed Central PMCID: PMC2607035. 18779055 PMC2607035

[13] Chen GH, Reddy RC, Newstead MW, Tateda K, Kyasapura BL, Standiford TJ. Intrapulmonary TNF gene therapy reverses sepsis-induced suppression of lung antibacterial host defense. Journal of immunology. 2000;165(11):6496–503. doi: 10.4049/jimmunol.165.11.6496 . 11086090

[14] Reddy RC, Chen GH, Newstead MW, Moore T, Zeng X, Tateda K, et al. Alveolar macrophage deactivation in murine septic peritonitis: role of interleukin 10. Infection and immunity. 2001;69(3):1394–401. doi: 10.1128/IAI.69.3.1394-1401.2001 ; PubMed Central PMCID: PMC98033. 11179304 PMC98033

[15] Deng JC, Cheng G, Newstead MW, Zeng X, Kobayashi K, Flavell RA, et al. Sepsis-induced suppression of lung innate immunity is mediated by IRAK-M. The Journal of clinical investigation. 2006;116(9):2532–42. doi: 10.1172/JCI28054 ; PubMed Central PMCID: PMC1550278. 16917541 PMC1550278

[16] Goya T, Abe M, Shimura H, Torisu M. Characteristics of alveolar macrophages in experimental septic lung. Journal of leukocyte biology. 1992;52(2):236–43. doi: 10.1002/jlb.52.2.236 . 1324290

[17] Jacobs RF, Kiel DP, Balk RA. Alveolar macrophage function in a canine model of endotoxin-induced lung injury. The American review of respiratory disease. 1986;134(4):745–51. doi: 10.1164/arrd.1986.134.4.745 . 3532887

[18] Harris SE, Nelson S, Astry CL, Bainton BG, Summer WR. Endotoxin-induced suppression of pulmonary antibacterial defenses against Staphylococcus aureus. The American review of respiratory disease. 1988;138(6):1439–43. doi: 10.1164/ajrccm/138.6.1439 . 3059896

[19] Munford RS, Weiss JP, Lu M. Biochemical Transformation of Bacterial Lipopolysaccharide by acyloxyacyl hydrolase reduces host injury and promotes recovery. The Journal of biological chemistry. 2020;295(51):17842–51. doi: 10.1074/jbc.REV120.015254 33454018 PMC7762960

[20] Munford R, Lu M, Varley A. Chapter 2: Kill the bacteria…and also their messengers? Advances in immunology. 2009;103:29–48. doi: 10.1016/S0065-2776(09)03002-8 ; PubMed Central PMCID: PMC2812913. 19755182 PMC2812913

[21] Lu M, Zhang M, Takashima A, Weiss J, Apicella MA, Li XH, et al. Lipopolysaccharide deacylation by an endogenous lipase controls innate antibody responses to Gram-negative bacteria. Nature immunology. 2005;6(10):989–94. doi: 10.1038/ni1246 . 16155573

[22] Lu M, Munford RS. The transport and inactivation kinetics of bacterial lipopolysaccharide influence its immunological potency in vivo. Journal of immunology. 2011;187(6):3314–20. doi: 10.4049/jimmunol.1004087 ; PubMed Central PMCID: PMC3169744. 21849675 PMC3169744

[23] Shao B, Lu M, Katz SC, Varley AW, Hardwick J, Rogers TE, et al. A host lipase detoxifies bacterial lipopolysaccharides in the liver and spleen. The Journal of biological chemistry. 2007;282(18):13726–35. doi: 10.1074/jbc.M609462200 . 17322564

[24] Zou B, Jiang W, Han H, Li J, Mao W, Tang Z, et al. Acyloxyacyl hydrolase promotes the resolution of lipopolysaccharide-induced acute lung injury. PLoS pathogens. 2017;13(6):e1006436. doi: 10.1371/journal.ppat.1006436 . 28622363 PMC5489216

[25] Shao B, Kitchens RL, Munford RS, Rogers TE, Rockey DC, Varley AW. Prolonged hepatomegaly in mice that cannot inactivate bacterial endotoxin. Hepatology. 2011;54(3):1051–62. doi: 10.1002/hep.24488 ; PubMed Central PMCID: PMC3188384. 21674560 PMC3188384

[26] Lu M, Varley AW, Munford RS. Persistently active microbial molecules prolong innate immune tolerance in vivo. PLoS pathogens. 2013;9(5):e1003339. doi: 10.1371/journal.ppat.1003339 ; PubMed Central PMCID: PMC3649966. 23675296 PMC3649966

[27] Barcik W, Boutin RCT, Sokolowska M, Finlay BB. The Role of Lung and Gut Microbiota in the Pathology of Asthma. Immunity. 2020;52(2):241–55. doi: 10.1016/j.immuni.2020.01.007 ; PubMed Central PMCID: PMC7128389. 32075727 PMC7128389

[28] McAleer JP, Kolls JK. Contributions of the intestinal microbiome in lung immunity. European journal of immunology. 2018;48(1):39–49. doi: 10.1002/eji.201646721 ; PubMed Central PMCID: PMC5762407. 28776643 PMC5762407

[29] Wypych TP, Wickramasinghe LC, Marsland BJ. The influence of the microbiome on respiratory health. Nature immunology. 2019;20(10):1279–90. doi: 10.1038/s41590-019-0451-9 . 31501577

[30] Enaud R, Prevel R, Ciarlo E, Beaufils F, Wieers G, Guery B, et al. The Gut-Lung Axis in Health and Respiratory Diseases: A Place for Inter-Organ and Inter-Kingdom Crosstalks. Frontiers in cellular and infection microbiology. 2020;10:9. doi: 10.3389/fcimb.2020.00009 ; PubMed Central PMCID: PMC7042389. 32140452 PMC7042389

[31] Qian G, Jiang W, Zou B, Feng J, Cheng X, Gu J, et al. LPS inactivation by a host lipase allows lung epithelial cell sensitization for allergic asthma. The Journal of experimental medicine. 2018;215(9):2397–412. doi: 10.1084/jem.20172225 ; PubMed Central PMCID: PMC6122967. 30021797 PMC6122967

[32] Balasubramanian D, Mathee K. Comparative transcriptome analyses of Pseudomonas aeruginosa. Human genomics. 2009;3(4):349–61. doi: 10.1186/1479-7364-3-4-361 ; PubMed Central PMCID: PMC2897818. 19706365 PMC2897818

[33] Lee YG, Jeong JJ, Nyenhuis S, Berdyshev E, Chung S, Ranjan R, et al. Recruited alveolar macrophages, in response to airway epithelial-derived monocyte chemoattractant protein 1/CCl2, regulate airway inflammation and remodeling in allergic asthma. American journal of respiratory cell and molecular biology. 2015;52(6):772–84. doi: 10.1165/rcmb.2014-0255OC ; PubMed Central PMCID: PMC4491131. 25360868 PMC4491131

[34] Schuijs MJ, Willart MA, Vergote K, Gras D, Deswarte K, Ege MJ, et al. Farm dust and endotoxin protect against allergy through A20 induction in lung epithelial cells. Science. 2015;349(6252):1106–10. doi: 10.1126/science.aac6623 . 26339029

[35] Thorley AJ, Ford PA, Giembycz MA, Goldstraw P, Young A, Tetley TD. Differential regulation of cytokine release and leukocyte migration by lipopolysaccharide-stimulated primary human lung alveolar type II epithelial cells and macrophages. Journal of immunology. 2007;178(1):463–73. doi: 10.4049/jimmunol.178.1.463 . 17182585

[36] Thorley AJ, Grandolfo D, Lim E, Goldstraw P, Young A, Tetley TD. Innate immune responses to bacterial ligands in the peripheral human lung—role of alveolar epithelial TLR expression and signalling. PloS one. 2011;6(7):e21827. doi: 10.1371/journal.pone.0021827 ; PubMed Central PMCID: PMC3137597. 21789185 PMC3137597

[37] Kopf M, Schneider C, Nobs SP. The development and function of lung-resident macrophages and dendritic cells. Nature immunology. 2015;16(1):36–44. doi: 10.1038/ni.3052 . 25521683

[38] Hussell T, Bell TJ. Alveolar macrophages: plasticity in a tissue-specific context. Nature reviews Immunology. 2014;14(2):81–93. doi: 10.1038/nri3600 . 24445666

[39] Quinton LJ, Mizgerd JP. Dynamics of lung defense in pneumonia: resistance, resilience, and remodeling. Annual review of physiology. 2015;77:407–30. doi: 10.1146/annurev-physiol-021014-071937 ; PubMed Central PMCID: PMC4366440. 25148693 PMC4366440

[40] Kinjyo I, Hanada T, Inagaki-Ohara K, Mori H, Aki D, Ohishi M, et al. SOCS1/JAB is a negative regulator of LPS-induced macrophage activation. Immunity. 2002;17(5):583–91. doi: 10.1016/s1074-7613(02)00446-6 . 12433365

[41] Nakagawa R, Naka T, Tsutsui H, Fujimoto M, Kimura A, Abe T, et al. SOCS-1 participates in negative regulation of LPS responses. Immunity. 2002;17(5):677–87. doi: 10.1016/s1074-7613(02)00449-1 . 12433373

[42] Guilliams M, Svedberg FR. Does tissue imprinting restrict macrophage plasticity? Nature immunology. 2021;22(2):118–27. doi: 10.1038/s41590-020-00849-2 . 33462453

[43] Kulikauskaite J, Wack A. Teaching Old Dogs New Tricks? The Plasticity of Lung Alveolar Macrophage Subsets. Trends in immunology. 2020;41(10):864–77. Epub 2020/09/09. doi: 10.1016/j.it.2020.08.008 ; PubMed Central PMCID: PMC7472979. 32896485 PMC7472979

[44] Cheng SC, Scicluna BP, Arts RJ, Gresnigt MS, Lachmandas E, Giamarellos-Bourboulis EJ, et al. Broad defects in the energy metabolism of leukocytes underlie immunoparalysis in sepsis. Nature immunology. 2016;17(4):406–13. doi: 10.1038/ni.3398 . 26950237

[45] Svedberg FR, Brown SL, Krauss MZ, Campbell L, Sharpe C, Clausen M, et al. The lung environment controls alveolar macrophage metabolism and responsiveness in type 2 inflammation. Nature immunology. 2019;20(5):571–80. doi: 10.1038/s41590-019-0352-y . 30936493 PMC8381729

[46] Woods PS, Kimmig LM, Meliton AY, Sun KA, Tian Y, O’Leary EM, et al. Tissue-Resident Alveolar Macrophages Do Not Rely on Glycolysis for LPS-induced Inflammation. American journal of respiratory cell and molecular biology. 2020;62(2):243–55. Epub 2019/08/31. doi: 10.1165/rcmb.2019-0244OC ; PubMed Central PMCID: PMC6993551. 31469581 PMC6993551

[47] Pereverzeva L, van Linge CCA, Schuurman AR, Klarenbeek AM, Ramirez Moral I, Otto NA, et al. Human alveolar macrophages do not rely on glucose metabolism upon activation by lipopolysaccharide. Biochim Biophys Acta Mol Basis Dis. 2022;1868(10):166488. Epub 2022/07/15. doi: 10.1016/j.bbadis.2022.166488 . 35835414

[48] Mosser DM. The many faces of macrophage activation. Journal of leukocyte biology. 2003;73(2):209–12. doi: 10.1189/jlb.0602325 . 12554797

[49] Roquilly A, Jacqueline C, Davieau M, Molle A, Sadek A, Fourgeux C, et al. Alveolar macrophages are epigenetically altered after inflammation, leading to long-term lung immunoparalysis. Nature immunology. 2020;21(6):636–48. doi: 10.1038/s41590-020-0673-x . 32424365

[50] Barclay AN, Brown MH. The SIRP family of receptors and immune regulation. Nature reviews Immunology. 2006;6(6):457–64. doi: 10.1038/nri1859 . 16691243

[51] Dobrovolskaia MA, Medvedev AE, Thomas KE, Cuesta N, Toshchakov V, Ren T, et al. Induction of in vitro reprogramming by Toll-like receptor (TLR)2 and TLR4 agonists in murine macrophages: effects of TLR "homotolerance" versus "heterotolerance" on NF-kappa B signaling pathway components. Journal of immunology. 2003;170(1):508–19. Epub 2002/12/24. doi: 10.4049/jimmunol.170.1.508 . 12496438

[52] Schuijt TJ, Lankelma JM, Scicluna BP, de Sousa e Melo F, Roelofs JJ, de Boer JD, et al. The gut microbiota plays a protective role in the host defence against pneumococcal pneumonia. Gut. 2016;65(4):575–83. doi: 10.1136/gutjnl-2015-309728 ; PubMed Central PMCID: PMC4819612. 26511795 PMC4819612

[53] Gray J, Oehrle K, Worthen G, Alenghat T, Whitsett J, Deshmukh H. Intestinal commensal bacteria mediate lung mucosal immunity and promote resistance of newborn mice to infection. Science translational medicine. 2017;9(376). doi: 10.1126/scitranslmed.aaf9412 ; PubMed Central PMCID: PMC5880204. 28179507 PMC5880204

[54] Brown RL, Sequeira RP, Clarke TB. The microbiota protects against respiratory infection via GM-CSF signaling. Nature communications. 2017;8(1):1512. doi: 10.1038/s41467-017-01803-x ; PubMed Central PMCID: PMC5688119. 29142211 PMC5688119

[55] Ichinohe T, Pang IK, Kumamoto Y, Peaper DR, Ho JH, Murray TS, et al. Microbiota regulates immune defense against respiratory tract influenza A virus infection. Proceedings of the National Academy of Sciences of the United States of America. 2011;108(13):5354–9. doi: 10.1073/pnas.1019378108 ; PubMed Central PMCID: PMC3069176. 21402903 PMC3069176

[56] Dessein R, Bauduin M, Grandjean T, Le Guern R, Figeac M, Beury D, et al. Antibiotic-related gut dysbiosis induces lung immunodepression and worsens lung infection in mice. Critical care. 2020;24(1):611. doi: 10.1186/s13054-020-03320-8 ; PubMed Central PMCID: PMC7574210. 33076936 PMC7574210

[57] Mason CM, Dobard E, Summer WR, Nelson S. Intraportal lipopolysaccharide suppresses pulmonary antibacterial defense mechanisms. The Journal of infectious diseases. 1997;176(5):1293–302. doi: 10.1086/514125 . 9359731

[58] Whitsett JA, Alenghat T. Respiratory epithelial cells orchestrate pulmonary innate immunity. Nature immunology. 2015;16(1):27–35. doi: 10.1038/ni.3045 ; PubMed Central PMCID: PMC4318521. 25521682 PMC4318521

[59] Bhattacharya J, Westphalen K. Macrophage-epithelial interactions in pulmonary alveoli. Seminars in immunopathology. 2016;38(4):461–9. doi: 10.1007/s00281-016-0569-x ; PubMed Central PMCID: PMC5018989. 27170185 PMC5018989

[60] Westphalen K, Gusarova GA, Islam MN, Subramanian M, Cohen TS, Prince AS, et al. Sessile alveolar macrophages communicate with alveolar epithelium to modulate immunity. Nature. 2014;506(7489):503–6. doi: 10.1038/nature12902 ; PubMed Central PMCID: PMC4117212. 24463523 PMC4117212

[61] Janelsins BM, Lu M, Datta SK. Altered inactivation of commensal LPS due to acyloxyacyl hydrolase deficiency in colonic dendritic cells impairs mucosal Th17 immunity. Proceedings of the National Academy of Sciences of the United States of America. 2014;111(1):373–8. doi: 10.1073/pnas.1311987111 ; PubMed Central PMCID: PMC3890863. 24344308 PMC3890863

[62] Katz SS, Weinrauch Y, Munford RS, Elsbach P, Weiss J. Deacylation of lipopolysaccharide in whole Escherichia coli during destruction by cellular and extracellular components of a rabbit peritoneal inflammatory exudate. The Journal of biological chemistry. 1999;274(51):36579–84. doi: 10.1074/jbc.274.51.36579 . 10593958

[63] Lu M, Zhang M, Kitchens RL, Fosmire S, Takashima A, Munford RS. Stimulus-dependent deacylation of bacterial lipopolysaccharide by dendritic cells. The Journal of experimental medicine. 2003;197(12):1745–54. doi: 10.1084/jem.20030420 ; PubMed Central PMCID: PMC2193946. 12810692 PMC2193946

[64] Staab JF, Ginkel DL, Rosenberg GB, Munford RS. A saposin-like domain influences the intracellular localization, stability, and catalytic activity of human acyloxyacyl hydrolase. The Journal of biological chemistry. 1994;269(38):23736–42. . 8089145

[65] Feulner JA, Lu M, Shelton JM, Zhang M, Richardson JA, Munford RS. Identification of acyloxyacyl hydrolase, a lipopolysaccharide-detoxifying enzyme, in the murine urinary tract. Infection and immunity. 2004;72(6):3171–8. doi: 10.1128/IAI.72.6.3171-3178.2004 ; PubMed Central PMCID: PMC415693. 15155618 PMC415693

[66] Gioannini TL, Teghanemt A, Zhang D, Prohinar P, Levis EN, Munford RS, et al. Endotoxin-binding proteins modulate the susceptibility of bacterial endotoxin to deacylation by acyloxyacyl hydrolase. The Journal of biological chemistry. 2007;282(11):7877–84. doi: 10.1074/jbc.M605031200 . 17227775

[67] Han Y-H, Onufer EJ, Huang L-H, Sprung RW, Davidson WS, Czepielewski RS, et al. Enterically derived high-density lipoprotein restrains liver injury through the portal vein. Science. 2021;373(6553):eabe6729. doi: 10.1126/science.abe6729 The information is not complete. PMCID: PMC8478306 34437091 PMC8478306

[68] Smith PD, Suffredini AF, Allen JB, Wahl LM, Parrillo JE, Wahl SM. Endotoxin administration to humans primes alveolar macrophages for increased production of inflammatory mediators. Journal of clinical immunology. 1994;14(2):141–8. doi: 10.1007/BF01541347 . 8195316

[69] Fitting C, Dhawan S, Cavaillon JM. Compartmentalization of tolerance to endotoxin. The Journal of infectious diseases. 2004;189(7):1295–303. doi: 10.1086/382657 . 15031800

[70] Cavaillon JM, Annane D. Compartmentalization of the inflammatory response in sepsis and SIRS. Journal of endotoxin research. 2006;12(3):151–70. doi: 10.1179/096805106X102246 . 16719987

[71] Philippart F, Fitting C, Cavaillon JM. Lung microenvironment contributes to the resistance of alveolar macrophages to develop tolerance to endotoxin*. Critical care medicine. 2012;40(11):2987–96. doi: 10.1097/CCM.0b013e31825b8d57 . 22878679

[72] Ruggiero G, Andreana A, Utili R, Galante D. Enhanced phagocytosis and bactericidal activity of hepatic reticuloendothelial system during endotoxin tolerance. Infection and immunity. 1980;27(3):798–803. Epub 1980/03/01. doi: 10.1128/iai.27.3.798-803.1980 ; PubMed Central PMCID: PMC550842. 6991430 PMC550842

[73] Wheeler DS, Lahni PM, Denenberg AG, Poynter SE, Wong HR, Cook JA, et al. Induction of endotoxin tolerance enhances bacterial clearance and survival in murine polymicrobial sepsis. Shock. 2008;30(3):267–73. Epub 2008/01/17. doi: 10.1097/shk.0b013e318162c190 ; PubMed Central PMCID: PMC2754132. 18197145 PMC2754132

[74] Ariga SK, Abatepaulo FB, Melo ES, Velasco IT, Pinheiro da Silva F, de Lima TM, et al. Endotoxin tolerance drives neutrophil to infectious site. Shock. 2014;42(2):168–73. Epub 2014/03/29. doi: 10.1097/SHK.0000000000000175 . 24667625

[75] Yoshimura A, Naka T, Kubo M. SOCS proteins, cytokine signalling and immune regulation. Nature reviews Immunology. 2007;7(6):454–65. doi: 10.1038/nri2093 . 17525754

[76] Bourdonnay E, Zaslona Z, Penke LR, Speth JM, Schneider DJ, Przybranowski S, et al. Transcellular delivery of vesicular SOCS proteins from macrophages to epithelial cells blunts inflammatory signaling. The Journal of experimental medicine. 2015;212(5):729–42. doi: 10.1084/jem.20141675 ; PubMed Central PMCID: PMC4419346. 25847945 PMC4419346

[77] Piñeros Alvarez AR, Glosson-Byers N, Brandt S, Wang S, Wong H, Sturgeon S, McCarthy BP, Territo PR, Alves-Filho JC, Serezani CH. SOCS1 is a negative regulator of metabolic reprogramming during sepsis. JCI Insight. 2017 Jul 6;2(13):e92530. doi: 10.1172/jci.insight.92530 ; PMCID: PMC5499360. 28679957 PMC5499360

[78] Feng J, Jiang W, Cheng X, Zou B, Varley AW, Liu T, et al. A host lipase prevents lipopolysaccharide-induced foam cell formation. iScience. 2021;24(9):103004. doi: 10.1016/j.isci.2021.103004 ; PubMed Central PMCID: PMC8426562. 34522852 PMC8426562

[79] Ural BB, Yeung ST, Damani-Yokota P, Devlin JC, de Vries M, Vera-Licona P, et al. Identification of a nerve-associated, lung-resident interstitial macrophage subset with distinct localization and immunoregulatory properties. Science immunology. 2020;5(45). doi: 10.1126/sciimmunol.aax8756 . 32220976 PMC7717505

[80] Chakarov S, Lim HY, Tan L, Lim SY, See P, Lum J, et al. Two distinct interstitial macrophage populations coexist across tissues in specific subtissular niches. Science. 2019;363(6432). Epub 2019/03/16. doi: 10.1126/science.aau0964 . 30872492

