# Supplementary material for: Acyloxyacyl hydrolase promotes pulmonary defense by preventing alveolar macrophage tolerance
Source: PLoS Pathog. 2023 Jul 27;19(7):e1011556. doi: 10.1371/journal.ppat.1011556 (PMC10409266; doi:10.1371/journal.ppat.1011556)
Supplement: S1 Fig — (DOCX) [file ppat.1011556.s001.docx]

**S1 Fig. *Aoah^-/-^* mice have reduced KC secretion and neutrophil recruitment after *Pseudomonas aeruginosa* infection.**

(A) *Aoah^+/+^* and *Aoah^−/−^* mice were instilled i.n. with 3 × 10^6^ PA. Control mice received PBS i.n. Five h later, the concentrations of inflammatory cytokines and chemokines in BALF were determined using ELISA.

(B) Cells in BALF were counted and analyzed using FACS.

(A, B) Data were combined from 2 experiments. n = 3 – 6. Mann-Whitney test was used. *, P < 0.05; **, P < 0.01.
