# Supplementary material for: Acyloxyacyl hydrolase promotes pulmonary defense by preventing alveolar macrophage tolerance
Source: PLoS Pathog. 2023 Jul 27;19(7):e1011556. doi: 10.1371/journal.ppat.1011556 (PMC10409266; doi:10.1371/journal.ppat.1011556)
Supplement: S2 Fig — (DOCX) [file ppat.1011556.s002.docx]

**S2 Fig. After exposure to LPS, AMs and AECs express different cytokines/chemokines.**

*Aoah^+/+^* mice were instilled with 10 μg LPS i.n. Five h later, AMs in BALF were allowed to adhere to plastic plates. CD45^-^CD326^+^ AECs were sorted using MACS. The mRNA abundance of inflammatory cytokines and chemokines were measured using real-time PCR. Data were combined from 2 – 3 experiments, n = 5 – 10. Mann-Whitney test was used. **, P < 0.01; ***, P < 0.001.
