# Supplementary material for: Acyloxyacyl hydrolase promotes pulmonary defense by preventing alveolar macrophage tolerance
Source: PLoS Pathog. 2023 Jul 27;19(7):e1011556. doi: 10.1371/journal.ppat.1011556 (PMC10409266; doi:10.1371/journal.ppat.1011556)
Supplement: S3 Fig — (DOCX) [file ppat.1011556.s003.docx]

**S3 Fig. After LPS i.n. instillation, *Aoah^-/-^* mouse lungs have reduced neutrophil proportion.**

Mice were instilled i.n. with 10 μg LPS or PBS. Five h later, the lung tissues were digested and single cell suspensions were prepared for FACS analysis.

(A) The gating strategy. Lymphocytes were excluded by gating out FSC^lo^SSC^lo^ cells. Myeloid immune cells (CD45^+^) were identified: CD11b^+^Ly6G^+^ neutrophils, Ly6G^-^CD11b^lo^CD11c^+^SiglecF^+^ alveolar macrophages (AMs), Ly6G^-^CD11b^+^SiglecF^+^ eosinophils, Ly6G^-^CD11b^+^CD11c^+^MHCII^+^ dendritic cells (DCs), Ly6G^-^CD11b^+^Ly6C^hi^ monocytes, Ly6G^-^CD11b^+^Ly6C^hi^CD64^+^ mono-macrophages (monocyte-derived macrophages), and Ly6G^-^CD11b^+^Ly6C^-^CD64^+^ interstitial macrophages (IMs).

(B) The frequency of each population was shown. Data were combined from 2 – 3 experiments. n = 4 – 9. Mann-Whitney test was used. ***, P < 0.001.
