# Supplementary material for: Acyloxyacyl hydrolase promotes pulmonary defense by preventing alveolar macrophage tolerance
Source: PLoS Pathog. 2023 Jul 27;19(7):e1011556. doi: 10.1371/journal.ppat.1011556 (PMC10409266; doi:10.1371/journal.ppat.1011556)
Supplement: S4 Fig — (DOCX) [file ppat.1011556.s004.docx]

**S4 Fig. *Aoah^-/-^* mouse blood or bone marrow monocytes are not tolerant.**

*Aoah^+/+^* or *Aoah^-/-^* mouse blood and bone marrow cells were stimulated with 10 ng/ml LPS in the presence of ER-Golgi protein trafficking inhibitor brefeldin A.

1. Six h later, the intracellular IL-6 and TNF-α of CD11b^+^Ly6C^hi^ monocytes were determined using FACS.
2. The proportions of TNF-α, IL-6 double positive cells of monocytes were calculated. Data were combined from 2 – 4 experiments. n = 4 – 12. Mann-Whitney test was used.
