# Supplementary material for: Acyloxyacyl hydrolase promotes pulmonary defense by preventing alveolar macrophage tolerance
Source: PLoS Pathog. 2023 Jul 27;19(7):e1011556. doi: 10.1371/journal.ppat.1011556 (PMC10409266; doi:10.1371/journal.ppat.1011556)
Supplement: S1 Table — (DOCX) [file ppat.1011556.s005.docx]

**S1 Table. Primers used for qPCR**

| Mouse gene symbols | Forward primer sequence | Reverse primer sequence |
| --- | --- | --- |
| Actin | 5’-GGCTGTATTCCCCTCCATCG-3’ | 5’-CCAGTTGGTAACAATGCCATGT-3’ |
| IL-6 | 5’-ATCGTGGAAATGAGAAAAGAGTTGT-3’ | 5’-AAGTGCATCATCGTTGTTCATACA-3’ |
| TNF-α | 5’-CATCTTCTCAAAATTCGAGTGACAA-3’ | 5’- TCAGCCACTCCAGCTGCTC-3’ |
| MIP-2 (CXCL2/3) | 5’-AGCTACATCCCACCCACACAG-3’ | 5’-AAAGCCATCCGACTGCATCT-3’ |
| KC (CXCL1) | 5’-CAAGAACATCCAGAGCTTGAAGGT-3’ | 5’-GTGGCTATGACTTCGGTTTGG-3’ |
| IRAK-M | 5’-TCCCACCTGAGGTGAAGCAT-3’ | 5’-TGTGACATTGGCTGGTTCCA-3’ |
| A20 | 5’-CTCAGAACCAGAGATTCCATGAAG-3’ | 5’-ACCTGTGTAGTTCGAGGCATGTC-3’ |
| SHIP | 5’- TCAGCCATATCTGCACTGACAAC-3′ | 5’-ACTCCCACTGCTCCCTTGTTT-3’ |
| SOCS1 | 5’- CCGTGGGTCGCGAGAAC-3’ | 5’-AGGAACTCAGGTAGTCACGGAGTA-3’ |
| MCP-1 (CCL2) | 5’-TTAAAAACCTGGATCGGAACCAA-3’ | 5’-GCATTAGCTTCAGATTTACGGGT-3’ |
| GM-CSF | 5’-GGCCTTGGAAGCATGTAGAGG-3’ | 5’- GGAGAACTCGTTAGAGACGACTT -3’ |
| CCL20 | 5’-GCCTCTCGTACATACAGACGC-3’ | 5’-CCAGTTCTGCTTTGGATCAGC-3’ |
| HIF-1α | 5’-GGGGAGGACGATGAACATCAA-3’ | 5’-GGGTGGTTTCTTGTACCCACA-3’ |
| Glut-1 | 5’-CAGTTCGGCTATAACACTGGTG-3’ | 5’-GCCCCCGACAGAGAAGATG-3’ |
| LDHa | 5’- TGTCTCCAGCAAAGACTACTGT-3’ | 5’- GACTGTACTTGACAATGTTGGGA-3’ |
| H2-Aa | 5’-TCAGTCGCAGACGGTGTTTAT-3’ | 5’-GGGGGCTGGAATCTCAGGT-3’ |
| H2-Ab1 | 5’-AGCCCCATCACTGTGGAGT-3’ | 5’-GATGCCGCTCAACATCTTGC-3’ |
| H2-Eb1 | 5’-GCGGAGAGTTGAGCCTACG-3’ | 5’-CCAGGAGGTTGTGGTGTTCC-3’ |
| CIITA | 5’-TGCGTGTGATGGATGTCCAG-3’ | 5’-CCAAAGGGGATAGTGGGTGTC-3’ |
| H2-DMa | 5’-CTCGAAGCATCTACACCAGTG-3’ | 5’-TCCGAGAGCCCTATGTTGGG-3’ |
| H2-DMb1 | 5’-ACCCCACAGGACTTCACATAC-3’ | 5’-GGATACAGCACCCCAAATTCA-3’ |
